# Life stage and taxonomy the most important factors determining vertebrate stoichiometry: A meta‐analysis

**DOI:** 10.1002/ece3.9354

**Published:** 2022-10-01

**Authors:** Emily M. May, Rana W. El‐Sabaawi

**Affiliations:** ^1^ Department of Biology University of Victoria Victoria British Columbia Canada

**Keywords:** ecological stoichiometry, elemental content, meta‐analysis, nitrogen, ontogeny and development, phosphorus, vertebrates

## Abstract

Whole‐body elemental composition is a key trait for determining how organisms influence their ecosystems. Using mass‐balance, ecological stoichiometry predicts that animals with higher concentrations of element X will selectively retain more X and will recycle less X in their waste than animals with lower X concentrations. These animals will also store high quantities of X during their lives and after their deaths (prior to full decomposition). Vertebrates may uniquely impact nutrient cycling because they store high quantities of phosphorus (P) in their bones. However, vertebrates have diverse body forms and invest variably in bone. Current analyses of vertebrate elemental content predominately evaluate fishes, typically neglecting other vertebrates and leaving much of the diversity unexplored. We performed a systematic review and identified 179 measurements of whole‐body percent phosphorus (%P), percent nitrogen (%N), and N to P ratio (N:P) from 129 unique species of non‐fish vertebrates (amphibians: 39 species; reptiles: 19 species; birds: 27 species; mammals: 46 species). We found that %P (mean: 1.94%; SD [standard deviation] = 0.77) and N:P (mean: 12.52) varied with taxonomy and life stage, while %N (mean: 10.51%; SD = 3.25) varied primarily with taxonomy. Habitat, diet, and size had small and inconsistent effects in different groups. Our study highlights two research gaps. Life stage, which is frequently neglected in stoichiometric studies, is an important factor determining vertebrate %P. Furthermore, amphibians dominate our dataset, while other vertebrate taxa are poorly represented in the current literature. Further research into these neglected vertebrate taxa is essential.

## Graphical abstract

We performed a systematic review evaluating how vertebrates vary in %N, %P, and N:P. We found that taxonomy and life stage were the best predictors overall.

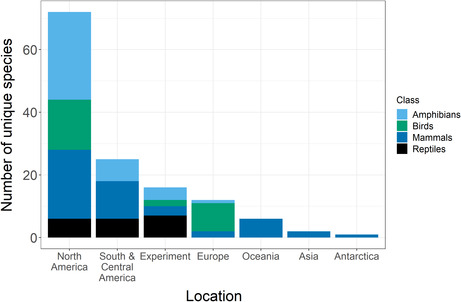

## 1 INTRODUCTION

Whole‐body elemental content reveals how animals have accumulated and stored nutrients and how they may retain or redistribute these nutrients (Sterner & Elser, 2002; Vanni et al., 2002; Vanni & McIntyre, 2016). In ecological stoichiometry, researchers can use mass‐balance to predict how an animal's whole‐body elemental content affects elemental uptake and release, assuming body elemental content remains within certain bounds (i.e. stoichiometric homeostasis with balanced growth; Sterner & Elser, 2002). Most current research in this area focuses on nitrogen (N) and phosphorus (P) (and their relative quantity: N:P), given their importance in nutrient limitation regimes (Sterner & Elser, 2002). Organisms vary in %N, %P, and N:P depending on their investment in both biomolecules and functional tissues (see Table 1; Sterner & Elser, 2002); thus, whole body elemental content is also useful for understanding functional trait investment (Jeyasingh et al., 2014). For example, the growth rate hypothesis posits that organisms with high growth rate will have relatively high %P and low N:P because they invest heavily in P‐rich ribosomal RNA (DeMott, 2003; Demott & Pape, 2005; Elser et al., 2003).

**TABLE 1:** Some of the most abundant sources of nitrogen and phosphorus in animal bodies

Element	Common biomolecules and tissues
Nitrogen	Proteins Nucleic acids Muscle Bone Keratin
Phosphorus	Nucleic acids Phospholipids Bone

Overall, ecological stoichiometry predicts that animals with higher concentrations of element “X” have higher X demand and so will retain more X from their diets and release less in their wastes (McIntyre & Flecker, 2010; Sterner & Elser, 2002). After death, those with high %X in their carcasses will recycle high quantities of X back into the ecosystem (complete decomposition) or will become a permanent store of X (incomplete decomposition) (Boros, Takács, et al., 2015; McIntyre & Flecker, 2010). Through these processes, animals alter nutrient supply rates and ratios, potentially enhancing or relaxing nutrient limitation regimes (Sterner & Elser, 2002). Thus, whole‐body elemental content is an important metric for assessing how animals impact their environments.

Vertebrates, often richer in N and P than other organisms (Evans‐White et al., 2005; McIntyre & Flecker, 2010; Vanni & McIntyre, 2016), are unique in ecological stoichiometry. Due to their relatively long lives and large bodies, vertebrates may act as large, long‐term stores of essential elements. During their lives, they recycle nutrients from their diet but retain considerable N and P within their bodies (Kitchell et al., 1979; Sterrett et al., 2015). After their deaths, vertebrate soft tissues usually fully decompose (Boros, Takács, et al., 2015; Subalusky et al., 2017) providing a rich supply of nutrients and carbon to the ecosystem. However; bone, a tissue unique to vertebrates, is not only uniquely rich in P (and in calcium and magnesium), but also resists decay (Pasteris et al., 2008; Subalusky et al., 2017). This may lead to permanent P sequestration in vertebrate carcasses or to a gradual release of P back into the ecosystem (a longer‐term nutrient source).

The importance of vertebrate‐driven nutrient cycling cannot be understated. Currently, however, most vertebrate groups are at risk (Barnosky et al., 2011; Hoffmann et al., 2010; Moritz & Agudo, 2013). These progressive losses will strongly impact how nutrients are stored and recycled (Lovich et al., 2018; Wenger et al., 2019). For example, Wenger et al. (2019) estimated historical inputs of vertebrate carcasses and found that vertebrate declines will strongly impact nutrient recycling, as both mass vertebrate deaths (e.g. mass deaths of migrating wildebeest or salmon provide nutrients for aquatic and terrestrial systems; Cederholm et al., 1999; Subalusky et al., 2017) and continuous autochthonous vertebrate deaths currently provide substantial nutrients to diverse ecosystems. Similarly, mass amphibian extinctions may change nutrient transfer between aquatic and terrestrial ecosystems (Fritz & Whiles, 2018; Kiesecker et al., 2001). To understand both how vertebrates impact nutrient cycles and how this may change, we must understand how vertebrates vary in elemental content.

### 1.1 Vertebrate elemental content

Most research on the factors determining elemental content occurs in invertebrates, shrouding vertebrates in mystery. Nonetheless, investment in bone, a P‐rich and metabolically unique tissue causes vertebrates to show unique patterns of elemental investment and allocation. Fishes currently dominate vertebrate ecological stoichiometry (e.g. see McIntyre & Flecker, 2010; Vanni & McIntyre, 2016) because aquatic ecosystems dominate ecological stoichiometry, moreover elementally explicit aquaculture studies are increasingly common. Several previous reviews characterize the extensive elemental diversity of fishes. Compiling the %P and %N of 100 fish species, McIntyre and Flecker (2010) found that fishes ranged from 1.3% to 5.7% P and 6.7% to 13.2% N and showed greater variation in %P (coefficient of variation [cv]: 29.8%) than %N (cv: 29.8%). Within aquaculture studies, Benstead et al. (2014) reported that commercially raised fishes showed %P from 0.9% to 4.6%. Individual species also showed tremendous variation. For example, in a large study of threespine stickleback on Canada's west coast, Durston and El‐Sabaawi (2017) found that stickleback ranged from 7.0% to 12.2% N and 2.2% to 6.5% P, similar ranges to all 100 fish species examined by McIntyre and Flecker (2010). Given the diversity in fishes, we wanted to characterize the diversity present in all vertebrates.

Non‐fish vertebrates (i.e. amphibians, birds, reptiles, and mammals) are morphologically and physiologically variable and likely vary widely in elemental content. Additionally, terrestrial and amphibious lifecycles present unique challenges to animals, including relatively nutrient poor or indigestible plant foods for herbivores, exposure to extreme weather, and the enhanced impact of gravity on structural tissues. Furthermore, many land vertebrates or secondarily aquatic vertebrates have unique adaptations, such as flight and endothermy, that may considerably alter form and function. The magnitude and drivers of the variation present in non‐fish vertebrates remain relatively uncharacterized.

#### 1.1.1 Drivers of elemental diversity

Because of bone's P‐richness and uniquely low N:P, skeletal variation and its correlates may drive elemental patterns in vertebrates. Life stage, size, habitat, and diet are four core traits that influence the skeleton and so that may influence the elemental content.

Life stage drives elemental content in many organisms. In vertebrates, development may drive elemental changes in two directions. Since high growth rate increases %P and organisms decrease their growth rates as they age, organisms theoretically decrease in %P as they age (i.e. as their RNA:DNA ratio decreases) (Elser et al., 2003; Pilar Olivar et al., 2009). However, vertebrates mineralize their bones with age, and the bone mineral (hydroxyapatite) is high in %P (Pasteris et al., 2008). Thus, vertebrate bone mineralization may override the impact of decreased growth rate, increasing %P, and decreasing N:P as vertebrates grow older. In fishes, researchers have indeed found increased %P across development, suggesting that bone does override growth rate (Boros, Sály, et al., 2015; Pilati & Vanni, 2007).

Size, even when separated from life stage, may also impact elemental content. Structural tissue investment scales allometrically as larger organisms require relatively more support against gravity (Anderson et al., 1979). Larger vertebrates, therefore, should have higher %P and lower N:P than smaller vertebrates. However, this relationship is likely weaker in aquatic vertebrates because animals living in buoyant media require less structural tissue (Anderson et al., 1979) but can invest in bone for other reasons, such as defense or maintaining neutral buoyancy (Clifton et al., 2008; Stein, 1989). As such, vertebrates living in aquatic environments alternatively show very high bone mineral density (Clifton et al., 2008) or very low bone mineral density (Guglielmini et al., 2002; Powell et al., 2019) depending on their behavior and physiology.

Finally, since organisms must consume sufficient nutrients to build and maintain their bodies, diet is vital in ecological stoichiometry (Jeyasingh et al., 2014). However, animals have many strategies, both behavioral and physiological, to take up sufficient nutrients from even the poorest diets (e.g. Durston & El‐Sabaawi, 2019; German & Horn, 2006; Jeyasingh et al., 2014; Olsson et al., 2007). Therefore, even species feeding on high N:P diets (i.e. low %P diets) can maintain a high whole‐body %P and a low N:P ratio. Indeed, many herbivores, such as armored catfish and cervids, invest heavily in bone despite their low‐P diets (Hood et al., 2005; Moen et al., 1999; Price & Allen, 2004). Nevertheless; asindividuals on nutritionally deficient diets may have difficulty mineralizing bones or may resorb their bones, diet likely impacts intraspecific or intraindividual bone content (Benstead et al., 2014). Thus, the impact of diet may depend on taxonomic scale.

Our study also analyzes the overall impact of taxonomy on vertebrate elemental content. Although individual species have adapted to unique nutrient conditions, organismal stoichiometry usually has a strong taxonomic signal (Allgeier et al., 2015; Andrieux et al., 2021; McIntyre & Flecker, 2010). As different vertebrate groups show unique traits that likely influence elemental composition (e.g. hard keratin shells in turtles or flight in birds), our study incorporates taxonomy at the class and order levels.

### 1.2 Our study

We performed a systematic review and meta‐analysis to evaluate elemental content in non‐fish vertebrates. Our goals were to (1) estimate the mean %N, %P, and N:P of non‐fish vertebrates, (2) identify major causes of elemental variation, and (3) identify gaps in the current vertebrate stoichiometry literature. Although most sources do not account for bony trait variation, we base many of our predictions on how variables affect the skeleton. First, we predict that %P will increase with age (i.e. the impact of bone will override the impact of growth rate). Second, we predict that size will impact elemental content separately from life stage, with larger vertebrates having higher %P and lower N:P. We predict that this relationship will be weaker in aquatic vertebrates. Finally, we predict that diet will not appreciably affect elemental content. Additionally, we examine taxonomy at class and order levels to broadly characterize each major vertebrate group.

Contrasting other recent systematic reviews examining elemental content in vertebrates (Andrieux et al., 2021; McIntyre & Flecker, 2010), our study exclusively focuses on whole‐body nutrient content and is the first meta‐analysis to incorporate developmental stage as a determinant of elemental content in vertebrates (Table S1). Additionally, while Andrieux et al. (2021) use elemental measurements from individual organisms as data points in their models, we use weighted general linear models with mean elemental measurements. Ideally, our review combined with theirs will provide a thorough overview of vertebrate elemental content.

## 2 MATERIALS AND METHODS

### 2.1 Systematic review

**FIGURE 1:**
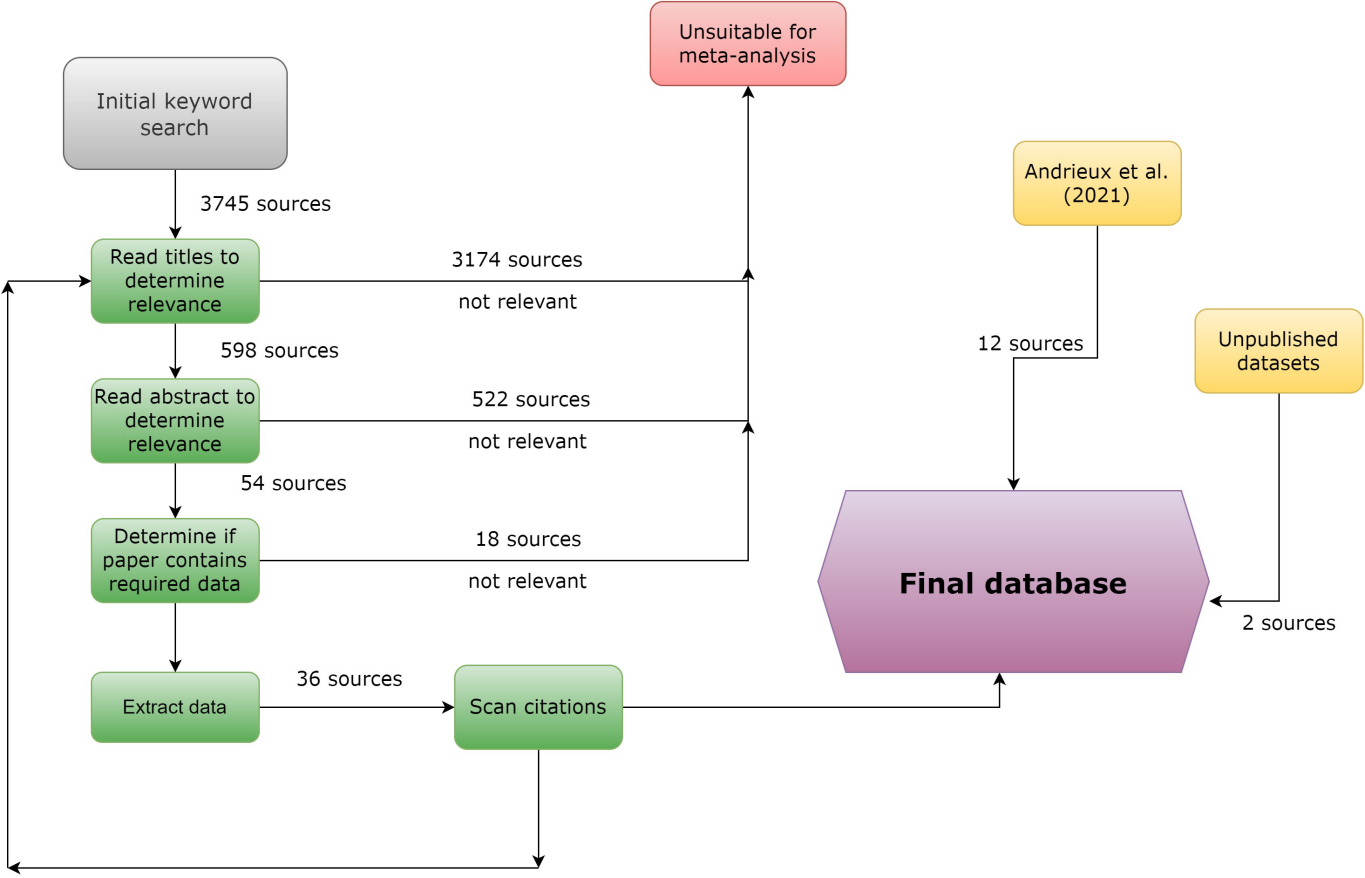
Summary of the systematic review process. Numbers indicate the number of unique sources successfully identified at each stage of the review process.

We performed a systematic review to locate sources measuring the N and/or P content of non‐fish vertebrates. On June 8, 2020, we searched the Web of Science database using the following search terms: TS=(stoich* OR element* content* OR element* composition OR nutri* content* OR phosphor* OR nitrogen OR nutri* composition OR nutrient ratio* OR element* ratio* OR chemical ratio*) AND (vertebrat* OR chordat* OR amphibian* OR lissamphib* OR reptil* OR bird* OR ave* OR mammal* OR frog* OR anura* OR urodel* OR salamander* OR snake* OR lizard* or squamat* OR crocod* OR turtle* OR tetrapod* OR tadpole*) AND (ecol* OR stoich*). We narrowed this search to include only potentially relevant categories of articles (Appendix S1). This search resulted in 3745 papers. We scanned the titles and abstracts of these papers to assess their relevance. Then, we read the abstracts of potentially relevant papers. To be included, papers had to (1) measure the whole‐body N and/or P of a non‐fish vertebrate, (2) be published after 1950 (papers before 1950 showed methodological ambiguity), and (3) provide a measure of variance for statistical weighting. When papers lacked sufficient information to calculate means or variances (e.g. data in boxplots), we contacted the authors. We did not use studies when we failed to acquire measures of variance. Then, we scanned the citation list of each relevant study to identify papers missed by the systematic review. Our final data source was Andrieux et al. (2021); we incorporated all unique values of whole‐body vertebrate %N, %P, and N:P available in their database. Although non peer‐reviewed zoo literature contained potentially relevant information, we did not include it due to methodological ambiguity (e.g. not always whole‐body measurements). We have summarized the systematic review process in Figure 1 and have listed our data sources in Appendix S2.

### 2.2 Data extraction

The primary response variables we analyzed were “whole‐body %P,” “whole‐body %N,” and “whole‐body N:P (molar ratio).” We initially extracted data on percent carbon (%C), C:P, and C:N; however, we ultimately did not analyze these data because %C varied considerably less than %P and %N (coefficient of variation of 8.9% compared to 39.7% for %P and 30.9% for %N) and because few studies provided measures of C:P or C:N (Figures S1 and S2). Additionally, %C alone is more vulnerable to seasonal and reproductive variation (e.g. changes in fat deposits), which are not always clearly accounted for. When available, we extracted taxonomic information, dry mass, life stage (larval amphibian/neonate amniote, juvenile, adult/paedomorph), general habitat (aquatic, terrestrial), generalized diet (herbivore, omnivore, carnivore), study location, whether animals were wild‐caught or lab‐reared, and whether the animals were eviscerated prior to analysis (Tables S2–S5). We determined diet either from the source itself or from conservation websites (e.g. audobon.org), and we classified animals as omnivores only if their intake of both animal and plant food was more than incidental. As most studies did not distinguish by sex, we could not extract sex data. When data were unavailable from the text, we extracted data from graphs using WebPlotDigitizer (https://apps.automeris.io/wpd/).

### 2.3 Statistical analysis

We analyzed data using weighted general linear models. To normalize residuals, we transformed response variables and dry mass measurements using the natural logarithm. We performed analyses within the entire dataset, within a subset including adults, within a subset including amphibians, and within a subset including dry mass estimates (note: amphibian data and dry mass data are presented in the Appendix S1). We evaluated adults separately to release us from inherent correlations between life stage and both diet and habitat. We did not assume a fixed true mean for any element but assumed that vertebrates naturally vary; thus, we weighted each %N and %P measurement by both the inverse of the study's variance and an estimate of between‐study variance (Appendix S1). Many studies lacked a variance estimate for N:P; therefore, we weighted N:P values by the inverse of the mean coefficient of variance for %N and %P, and we do not have true estimates for the weighted variance of N:P.

Within each data subset, we constructed weighted general linear models using the “dredge” function from the R package *MuMIn* and ranked the models using ΔAICc (Bartoń, 2020; R Core Team, 2021). Our models examine taxonomy (class, order, family), life stage, habitat type, and general diet (Table 2). We initially included “evisceration (yes/no)” and “field vs. laboratory study” as random effects. We removed “evisceration (yes/no)” because few studies eviscerated animals. We removed “field vs. laboratory study” because laboratory studies contained a greater proportion of sub‐adult measures than field studies, biasing this effect. Thus, our models incorporate fixed effects only. We assumed models with ΔAICc ≤ 2 were equivalent, and we averaged equivalent models (*model.avg* function from *MuMIn*) if multiple models had ΔAICc ≤ 2. If there was one best model, we presented only that model.

**TABLE 2:** Global models used (fixed effects only)

Response	Dataset	Global model
%P, %N, N:P	Entire dataset	~ class + order + life stage + diet + habitat
%P, %N, N:P	Adult dataset	~ class + order + diet + habitat

*Note*: Response variables were log_e_ (ln) transformed in models.

## 3 RESULTS

### 3.1 Systematic review

**FIGURE 2:**
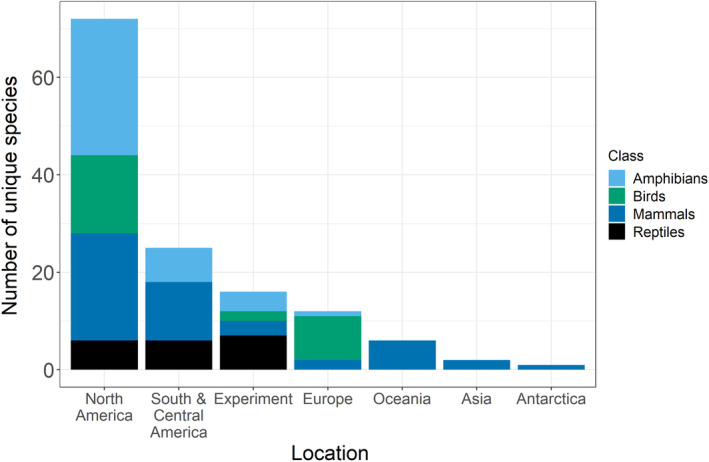
Geographical distribution of unique species measurements in our systematic review.

The systematic review identified 179 measurements from 50 sources, covering 129 unique species (Figure 2). Amphibians were the most studied class, with 76 total measurements from 39 unique species. Most studies were from the USA, while most other locations were poorly represented (Figure 2).

### 3.2 Entire dataset—statistical modeling

#### 3.2.1 Phosphorus

**FIGURE 3:**
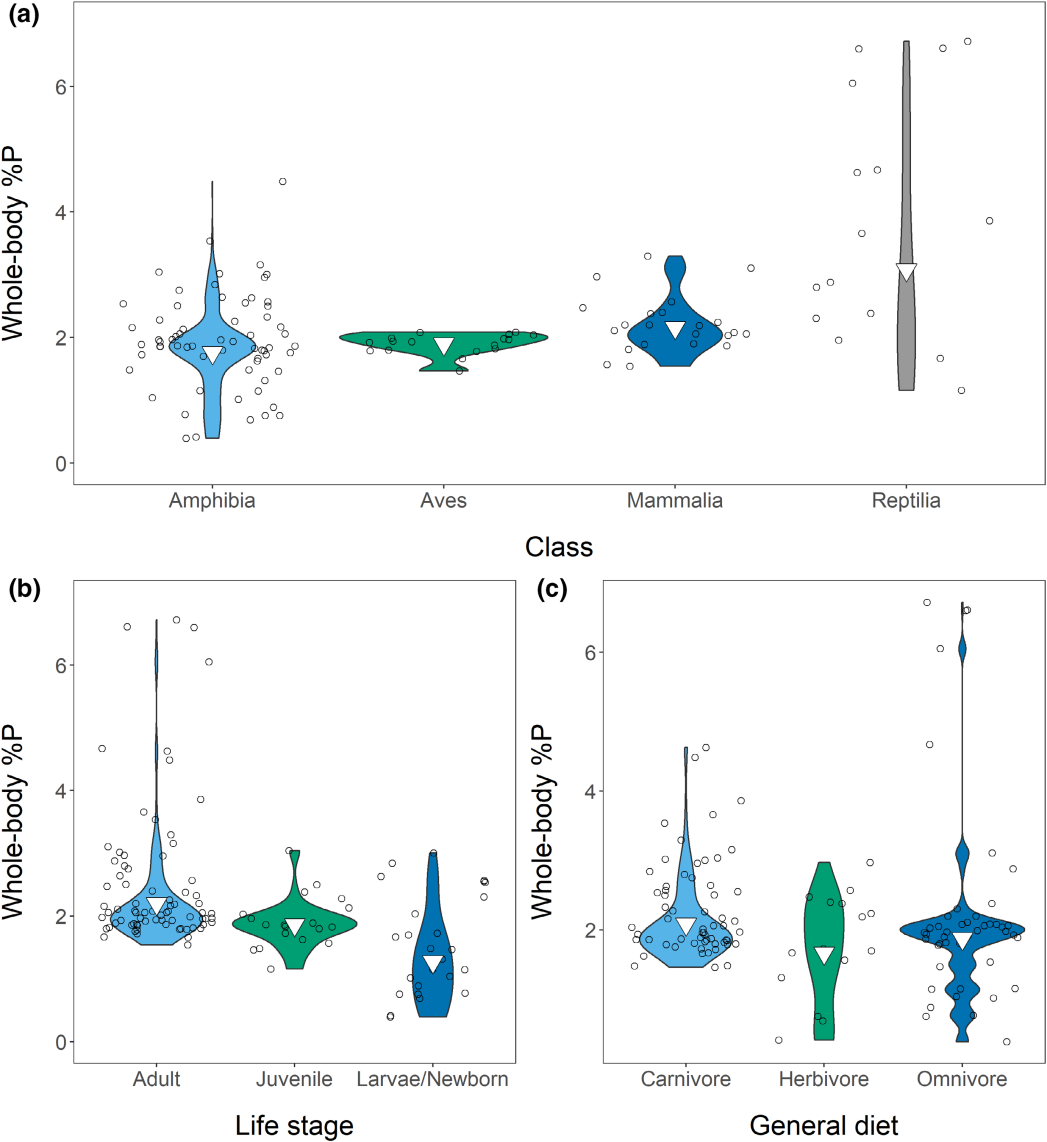
Effects of class (a), life stage (b), and diet (c) on whole‐body %P in our entire dataset. Inverted triangles show means, and points are jittered.

The weighted mean %P was 1.94% (variance [*s* ^2^] = 0.59; *n* = 120), and the model **%P ~ class + life stage + general diet** (Tables S6 and S7) best explained variation. Reptiles drove between‐class differences: reptiles had the highest %P (3.08%; *s* ^2^ = 3.17; *n* = 15), followed by mammals (2.15%; *s* ^2^ = 0.19; *n* = 22), birds (1.90%; *s* ^2^ = 0.34; *n* = 17), and amphibians (1.74%; *s* ^2^ = 0.41; *n* = 66) (Figure 3a). Life stage showed the predicted pattern: adults had the highest %P (2.20%; *s* ^2^ = 0.56; *n* = 76), followed by juveniles (1.86%; *s* ^2^ = 0.13; *n* = 19) and larvae/neonates (1.27%; *s* ^2^ = 0.47; *n* = 25) (Figure 3b). Amphibians affected this pattern disproportionately, as we had few sub‐adult measurements from amniotes (*n* = 7). Diet had a small but significant effect: carnivores had the highest %P (2.08%; *s* ^2^ = 0.30; *n* = 60), followed by omnivores (1.87%; *s* ^2^ = 0.87; *n* = 45) and herbivores (1.66%; *s* ^2^ = 0.56; *n* = 15) (Figure 3c). We visualized all factors in our Appendix S1 (Figures S1–S3).

#### 3.2.2 Nitrogen

**FIGURE 4:**
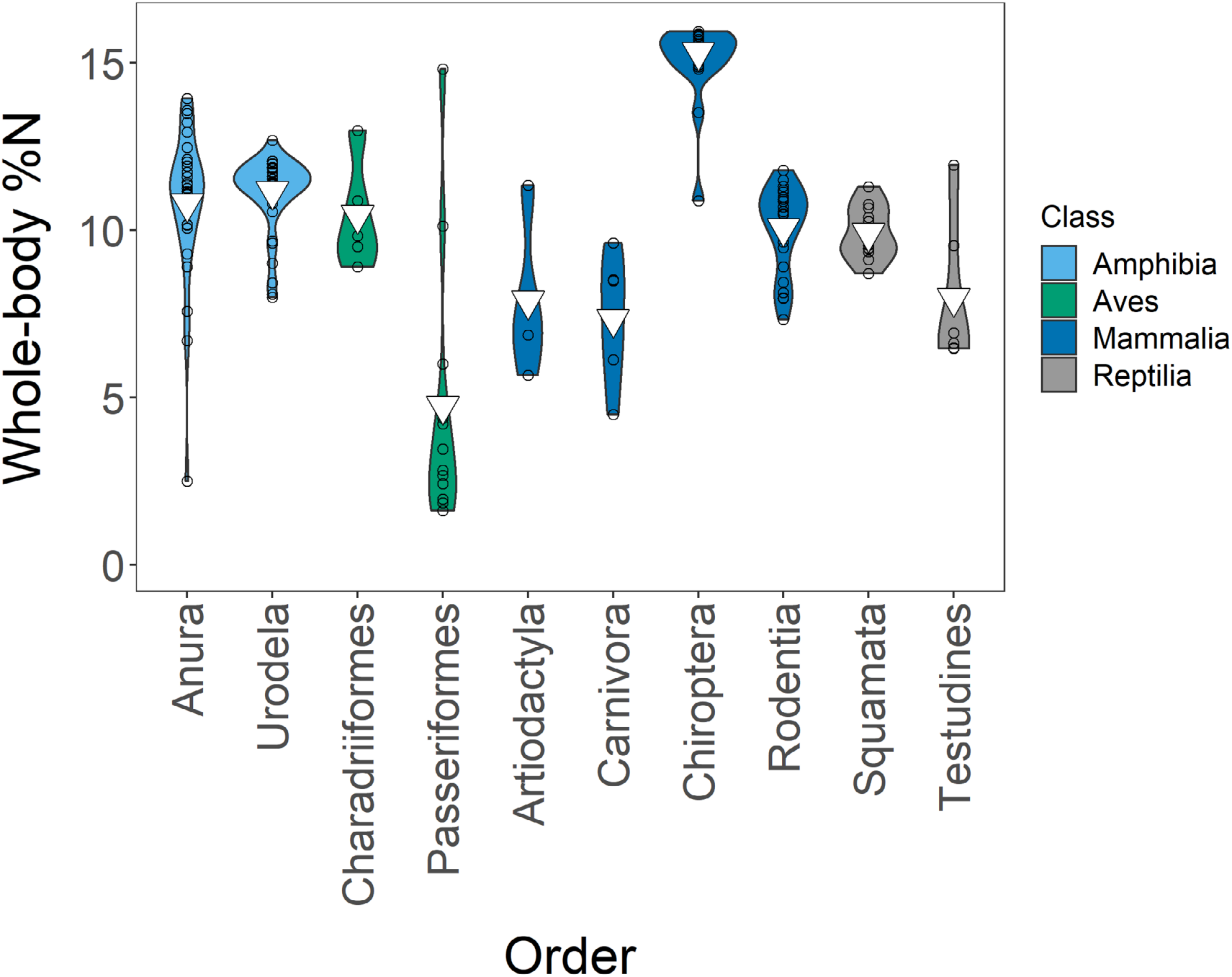
Relationships between order and whole‐body %N in our entire dataset. For clarity, we show only orders with *n* > 2. Inverted triangles show means.

**FIGURE 5:**
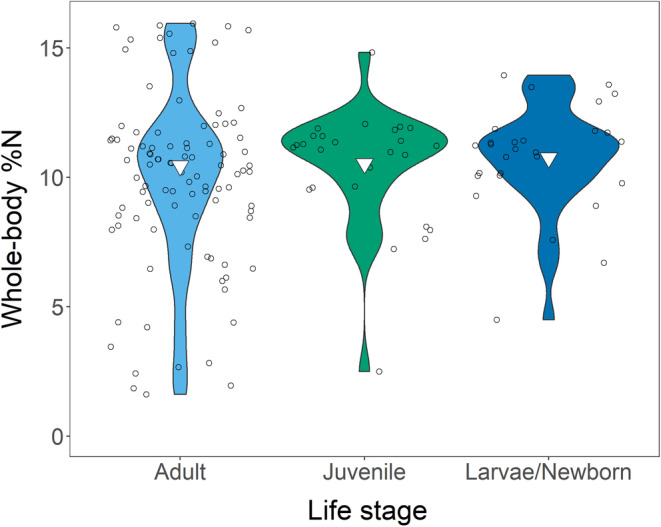
Relationship between vertebrate whole‐body %N and organismal life stage in our entire dataset. Points are jittered, and inverted triangles show means.

The weighted mean %N was 10.51% (*s* ^2^ = 10.54; *n* = 155). We had six equivalent models (ΔAICc ≤2): (1) **%N ~ order**, (2) **%N ~ class + order**, (3) **%N ~ order + life stage**, (4) **%N ~ class + order + life stage**, (5) **%N ~ order + life stage + habitat**, and (6) **%N ~ class + order + life stage + habitat** (Tables S8–S10). However, since order explained class effects, we averaged models 1, 3, and 5 only (Tables S9 and S10). Order had the greatest impact of all our variables: Order Chiroptera (15.32%; *s* ^2^ = 2.09; *n* = 19) and Order Passeriformes (4.75%; *s* ^2^ = 32.80; *n* = 12), which had the highest and lowest %N respectively, drove this effect (Figure 4). Neither the life stage nor the habitat significantly influenced %N (Figure 5, Figures S6 and S7). We visualized all factors in our Appendix S1 (Figures S4–S7).

#### 3.2.3 N:P

**FIGURE 6:**
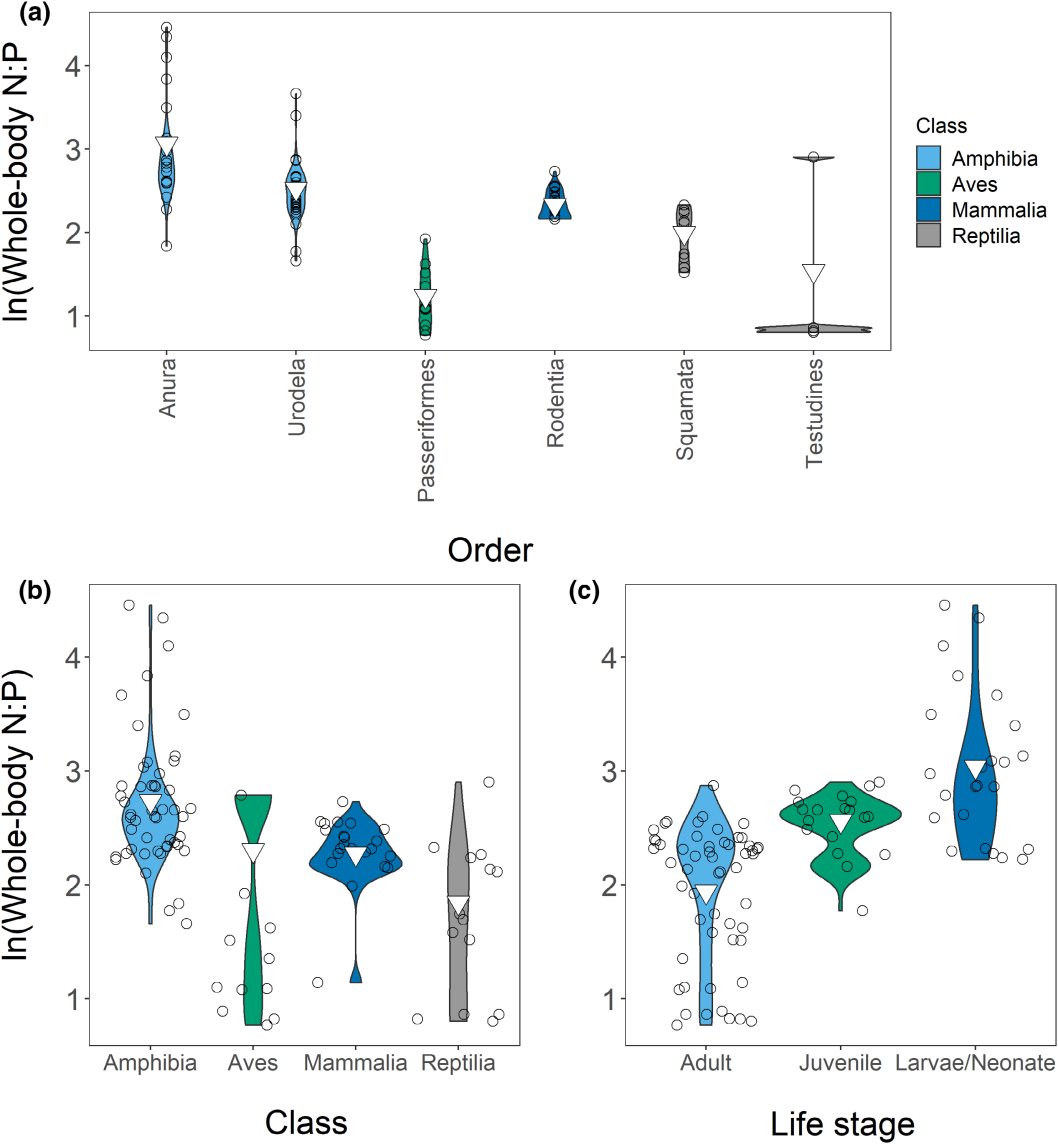
Relationships between class (a), order (b), and life stage (c) and whole‐body N:P. we have shown only orders with *n* > 2 in the order plot. Points are jittered in (b) and (c). Inverted triangles show weighted means.

The weighted mean N:P was 12.52 (range: 2.15–86.11; *n* = 98). We had six equivalent best models: (1) **N:P ~ order + diet**, (2) **N:P ~ class + order + diet**, (3) **N:P ~ order + life stage + diet**, (4) **N:P ~ class + order + life stage + diet**, (5) **N:P ~ order + life stage**, and (6) **N:P ~ class + order + life stage** (Tables S11–S13), and we averaged these models. Amphibians had a higher N:P (15.43; range = 5.26–86.11; *n* = 51) and a wider range than other classes. Birds (10.01; range = 2.16–16.22; *n* = 11) and mammals (9.70; range = 3.13–15.34; *n* = 22) had similar means and ranges, while reptiles (6.32; range = 2.23–18.23; *n* = 14) had the lowest N:P (Figure 6a). Within amphibians, Order Anura (21.36; range = 6.27–86.11; *n* = 23) and Order Urodela (12.48; range = 5.26–39.10; *n* = 28) had higher N:P than other orders, and this drove order relationships (Figure 6b). N:P decreased across developmental stages with larvae/neonates having the highest N:P (21.14; range = 9.23–86.11; *n* = 24) followed by juveniles (12.51; range = 5.88–18.23; *n* = 19) and adults (8.71; range = 2.16–17.68; *n* = 55) (Figure 6c). Taxonomic biases in life stage measurements (e.g. most sub‐adults were amphibians) may have impacted patterns. Note that, despite its statistical significance diet minimally impacted N:P. Overall, changes in %P rather than changes in %N drove N:P. We visualized all variables in our Appendix S1 (Figures S8–S10).

### 3.3 Adults only—statistical modeling

#### 3.3.1 Phosphorus

**FIGURE 7:**
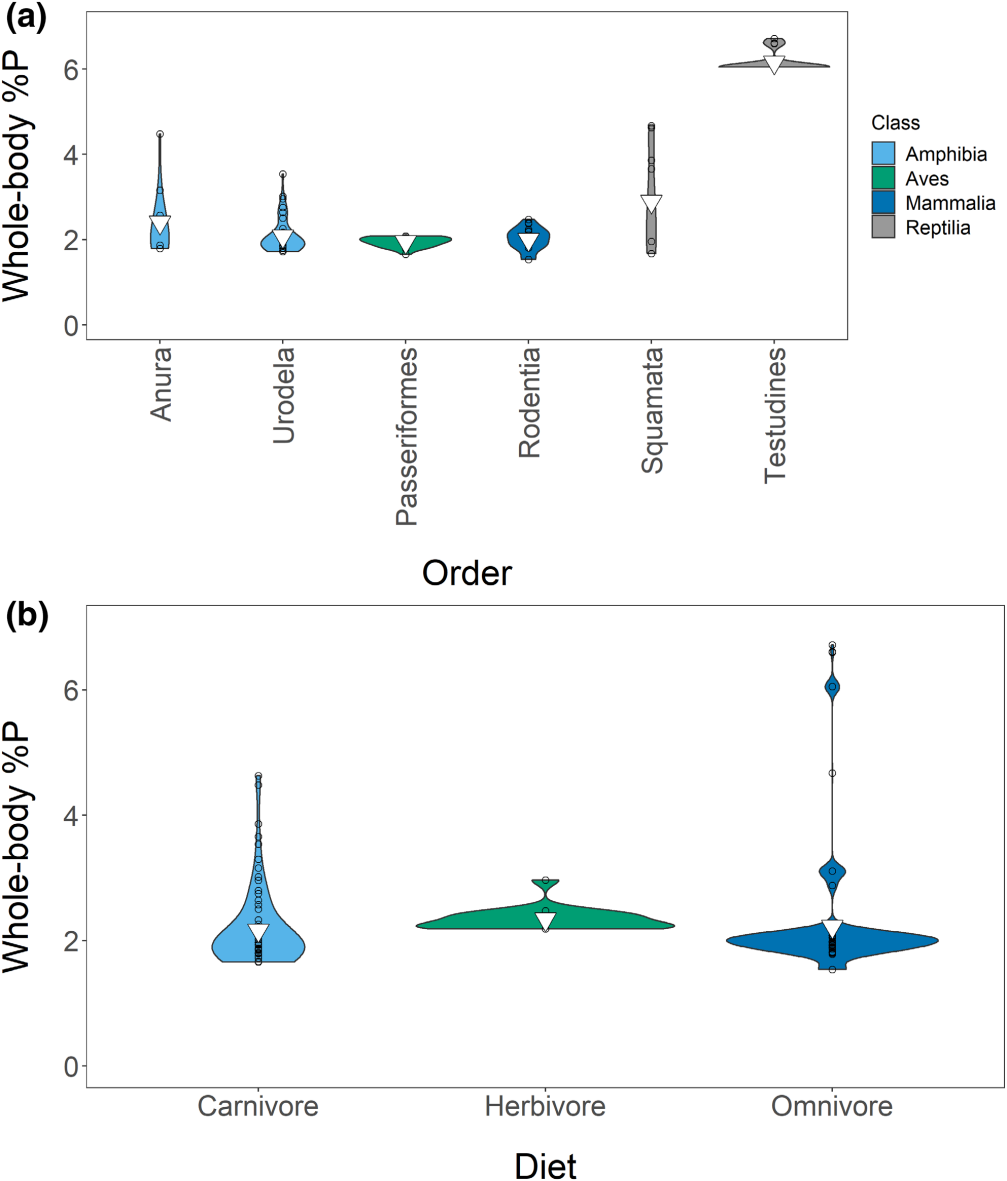
Effects of order (a) and diet (b) on whole‐body %P in adult vertebrates. We have shown only orders with *n* > 2. Inverted triangles show means.

The weighted mean %P for adults was 2.20% (*s* ^2^ = 0.56; *n* = 76). We had four top models: (1) **%P ~ order + general diet**, (2) **%P ~ class + order + general diet**, (3) **%P ~ order**, and (4) **%P ~ class + order** (Tables S14–S16). However, because order differences drove class effects, we averaged models 1 and 3 only (Figure 7, Tables S14–S16). As in our entire dataset, reptiles had the highest %P (3.76%; *s* ^2^ = 3.05; *n* = 12), while mammals (2.17%; *s* ^2^ = 0.21; *n* = 19), birds (1.95%; *s* ^2^ = 0.013; *n* = 16), and amphibians (2.11%; *s* ^2^ = 0.18; *n* = 29) had similar %P. Order Testudines (6.16%; *s* ^2^ = 0.055; *n* = 4) had higher %P than other orders and drove most order effects (Figure 7a). %P and diet showed a different relationship than they did in our whole dataset, but this relationship is statistically insignificant and weak. Herbivores (2.34%; *s* ^2^ = 0.035; *n* = 6) had the highest %P, followed by omnivores (2.22%; *s* ^2^ = 0.82; *n* = 32) and carnivores (2.17%; *s* ^2^ = 0.37; *n* = 38) (Figure 7b). Note that we analyzed few adult herbivores (*n* = 6).

#### 3.3.2 Nitrogen

**FIGURE 8:**
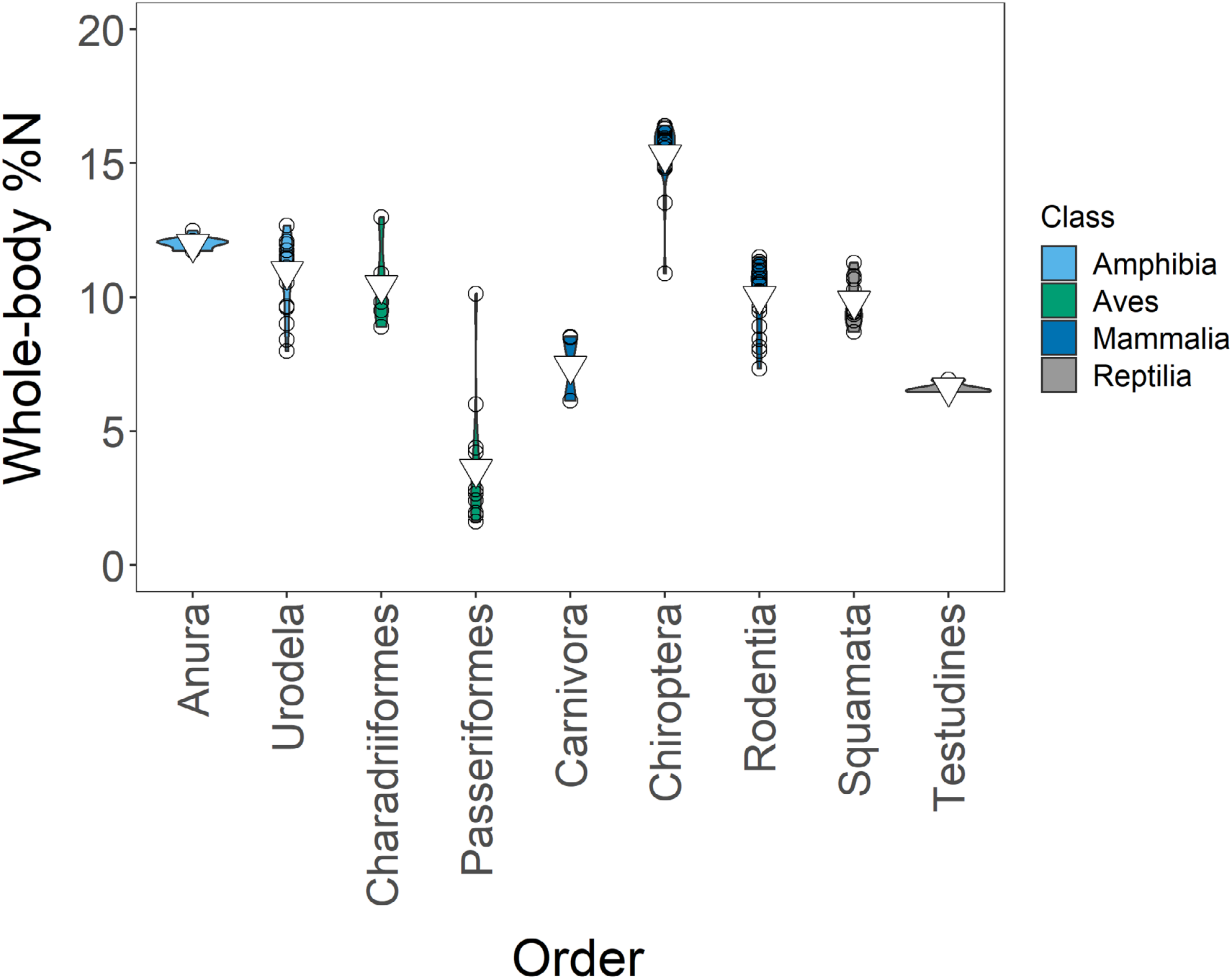
Relationships between order and whole‐body %N in adults. We have shown only orders with *n* > 2. Points are jittered. Inverted triangles show weighted means.

The mean %N for adults was 10.44% (*s* ^2^ = 14.11; *n* = 101), and as in our whole dataset the taxonomy best explained %N. Our best models were (1) **%N ~ order** and (2) **%N ~ class + order**, but since order differences explained class differences, we present model 1 only (Tables S17 and S18). Order Chiroptera (15.32%; *s* ^2^ = 2.09; *n* = 19) had the highest %N and Order Passeriformes had the lowest %N and the highest variance (4.75%; *s* ^2^ = 32.80; *n* = 12) (Figure 8). We have plotted class relationships in our Appendix S1.

#### 3.3.3 N:P

**FIGURE 9:**
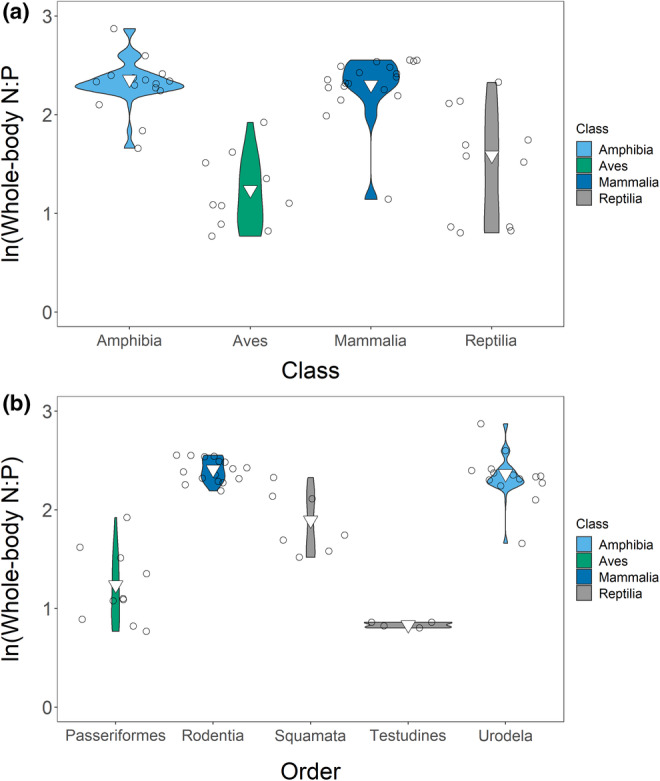
Relationships between class (a) and order (b) and whole‐body N:P in our adult‐only dataset. We have shown only orders with *n* > 2 in the order plot. Points are jittered. Inverted triangles show weighted means.

**FIGURE 10:**
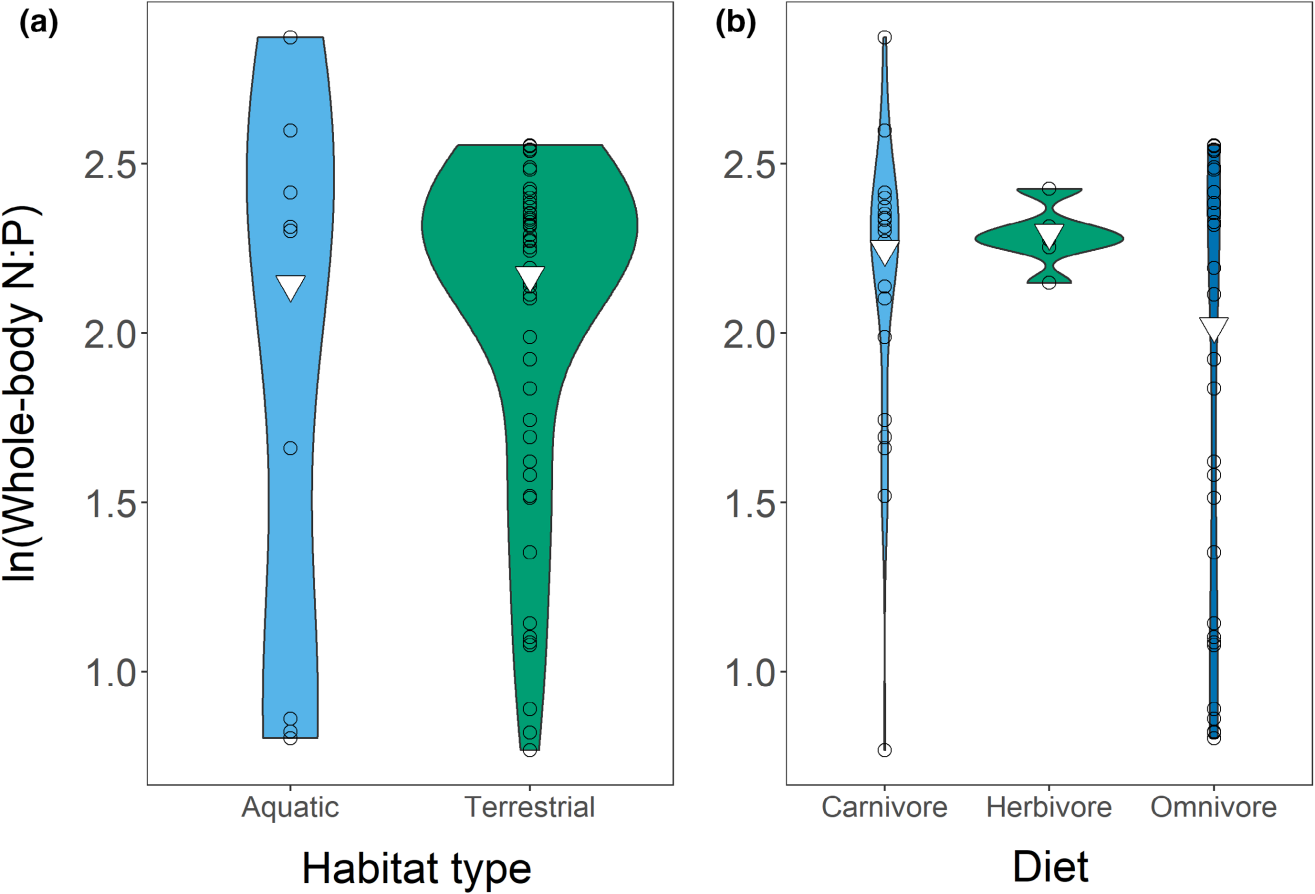
Relationships between habitat (a) and diet (b) and whole‐body N:P in our adult‐only dataset. Inverted triangles show weighted means.

The weighted mean N:P for adults was 8.71 (range = 2.16–17.68; *n* = 55). Taxonomy drove variation. Our four top models were: (1) **N:P ~ order + diet**, (2) **N:P ~ class + order + diet**, (3) **N:P ~ order + diet + habitat**, and (4) **N:P ~ class + order + diet + habitat** (Tables S19–S21). As variation in order explained variation in class we averaged models 1 and 3. We saw similar patterns in adults as in our entire dataset (Figure 9). Order Urodela (10.66; range = 5.26–17.68; *n* = 14) and Order Rodentia (11.14; range = 8.96–12.86; *n* = 16) had the highest N:P, while Order Passeriformes (3.46; range = 2.16–6.84; *n* = 10) and Order Testudines (2.30; range = 2.23–2.37; *n* = 4) had the lowest. This meant that adult amphibians (10.57; range = 5.26–17.68; *n* = 15) and adult mammals (10.02; range = 3.13–12.86; *n* = 19) showed high N:P, while adult reptiles (4.90; range = 2.23–10.26; *n* = 11) and adult birds (3.46; range = 2.16–6.84) showed lower N:P. Diet also affected N:P (Figure 10). Omnivores (7.54; range = 2.23–12.86; *n* = 29) had lower N:P than either carnivores (9.46; range = 2.15–17.68; *n* = 20) or herbivores (9.93; range = 8.58–11.31; *n* = 6); however, taxonomy likely confounded this. Habitat weakly influenced N:P despite its inclusion in the averaged model (Figure 10). Overall, increased %P caused decreased N:P, except in Order Passeriformes, which had low N:P and low %P.

## 4 DISCUSSION

With this meta‐analysis, we addressed two issues common in vertebrate elemental content studies: the emphasis on fishes and the lack of life stage data. Because aquatic nutrient recycling is well‐studied and fishes are frequently used in elementally focused aquaculture studies, they dominate the vertebrate stoichiometry literature. However, the diversity found in amphibians, reptiles, birds, and mammals uniquely impact the way vertebrates store and cycle nutrients. Understanding why non‐fish vertebrates vary elementally is thus essential for understanding nutrient storage in animal biomass. Understanding life stage is equally important, as ontogenetic differences reveal how animals accumulate nutrients over their lives and how they may impact nutrient cycles throughout their lives. Many vertebrates transition between habitats and diets as they mature (e.g. amphibians), making these relationships particularly interesting. Although some individual studies have evaluated how life stage impacts body stoichiometry (e.g. Boros, Sály, et al., 2015; Pilati & Vanni, 2007; Stephens et al., 2017), no meta‐analyses have yet evaluated how life stage influences body stoichiometry in vertebrates.

Given this, we had three goals. First, we wanted both to estimate the mean %P, %N, and N:P of non‐fish vertebrate groups and to characterize vertebrate elemental diversity. Second, we wanted to determine how ecological and organismal variables (especially life stage) contribute to elemental variation. Third, we wanted to identify the gaps present in the literature. Additionally, since Andrieux et al. (2021) recently published a meta‐analysis on heterotroph stoichiometry, we wanted both to compare our results to theirs, since we used different methodology (Table S1), and to evaluate how incorporating life stage can alter results.

We found that although taxonomy explains variation in %P, %N, and N:P, life stage only affected %P and N:P. This implies that age‐dependent bone mineralization mainly affects %P. Our other variables (diet, habitat, and size) had smaller effects. While diet explained some variation, its effects were inconsistent. Habitat did not show the predicted patterns, and size had no clear effect (Figures S15–S23).

### 4.1 Comparison to other studies

We compared our results to other large stoichiometric studies (Table 3). The mean %P, %N, and N:P of adult non‐fish vertebrates was comparable to adult Actinopterygiian fishes, as presented by both McIntyre and Flecker (2010) and Andrieux et al. (2021). Our class‐specific means for %P and %N were similar to Andrieux et al. (2021)'s, except for %P in reptiles (Table 3). However, vertebrates in their study generally showed higher N:P (Andrieux et al., 2021); this was potentially because their N:P dataset contained many amphibian measurements (321/408) and lacked life stage distinctions (Table S1). As amphibian larvae are high in N:P, this may have heavily influenced patterns in their data, explaining this discrepancy between our studies.

**TABLE 3:** Comparison of %P, %N, and N:P from our study (weighted means) and four other stoichiometric studies

Variable	Our study (^1^All adults; ^2^Adult amphibians; ^3^Adult reptiles; ^4^Adult mammals; ^5^Adult birds)	Our study (^1^All vertebrates; ^2^Amphibians; ^3^Reptiles; ^4^Mammals; ^5^Birds)	Andrieux et al. (2021)^a^ (^1^All non‐fish vertebrates; ^2^Amphibians; ^3^Reptiles; ^4^Mammals; ^5^Birds; ^5^Actinopterygii; ^6^Invertebrates; ^7^Microbes)	McIntyre and Flecker (2010) (Actinopterygii)	Evans‐White et al. (2005) (^1^Insects; ^2^Molluscs; ^3^Crustaceans)	Zhang and Elser (2017) (^1^Ectomycorrhizal fungi; ^2^Pathotrpohic fungi; ^3^Saprotrophic fungi)
%P	^1^2.20% (0.75)	^1^1.94% (0.77)	^1^2.36% (1.56)	2.9% (0.86)	^1^0.56% (0.18)	^1^0.58% (0.64)
	^2^2.11% (0.42)	^2^1.74% (0.64)	^2^1.87% (1.14)		^2^0.81% (0.13)	^2^6.90 (16.30)
	^3^3.76% (1.75)	^3^3.08% (1.78)	^3^5.13% (1.69)		^3^0.94% (0.12)	^3^1.28% (4.22)
	^4^2.17% (0.46)	^4^2.15% (0.44)	^4^2.27% (0.52)			
	^5^1.95% (0.11)	^5^1.90% (0.58)	^5^1.96% (0.23)			
			^6^3.09% (1.14)			
			^7^0.91% (0.57)			
			^8^0.74% (0.62)			
%N	^1^10.40% (3.76)	^1^10.51% (3.25)	^1^11.26% (2.65)	10.1% (1.21)	^1^10.0% (1.2)	^1^3.89% (1.10)
	^2^11.17% (1.39)	^2^10.99% (1.86)	^2^11.82% (1.76)		^2^9.6% (1.6)	^2^4.41% (1.88)
	^3^8.74% (2.22)	^3^9.18% (2.07)	^3^9.28% (2.69)		^3^7.4% (0.5)	^3^5.66% (9.01)
	^4^11.93% (3.52)	^4^11.52% (3.53)	^4^11.65% (3.10)			
	^5^6.12% (4.70)	^5^7.11% (3.53)	^5^8.97% (3.86)			
			^6^10.10% (1.38)			
			^7^9.60% (2.21)			
			^8^4.08% (2.18)			
N:P	^1^8.71	^1^12.52	^1^24.70 (29.47)	8.4 (2.52)	^1^46 (20)	^1^24.61 (18.28)
	^2^10.57	^2^15.43	^2^29.62 (31.40)		^2^27.3 (4.3)	^2^11.92 (7.55)
	^3^4.90	^3^6.32	^3^4.14 (3.20)		^3^17.5 (3.4)	^3^55.69 (106.69)
	^4^10.02	^4^9.70	^4^10.31 (1.84)			
	^5^3.46	^5^10.01	^5^5.47 (4.87)			
			^6^8.59 (4.32)			
			^7^30.40 (23.97)			
			^8^16.94 (13.62)			

*Note*: Standard deviations in parentheses.

a Calculated directly from available data.

We looked at two large studies by Evans‐White et al. (2005) and by Zhang and Elser (2017), which examined invertebrates and fungi, respectively. Vertebrates generally had higher %P, higher %N, and lower N:P than these taxa, excepting pathotrophic fungi, which had exceptionally high %P (Zhang & Elser, 2017; Table 3). This suggests that vertebrates are indeed a rich nutrient source.

### 4.2 Taxonomy

Taxonomy drives elemental content in diverse groups, including fishes (e.g. McIntyre & Flecker, 2010), insects (e.g. Fagan et al., 2002), and benthic macroinvertebrates (e.g. Evans‐White et al., 2005). In our study, order drove most taxonomic relationships, revealing substantial intraclass variation. Bone differences likely drove taxonomic differences. For example, anuran larvae had the lowest %P (and likely the lowest bony investment) due to their life stage, habitat, and highly cartilaginous skeletons, while adult testudines had the highest %P due to the extensive bony structures associated with their shells (Iverson, 1984; Sterrett et al., 2015). Contrastingly, differences in muscle and fat likely caused most %N variation; muscle accumulation directly increases %N, while fat accumulation increases %C, potentially increasing C:N and thus indirectly decreasing %N. For example, phocids (seals) that show high fat accumulation showed lower %N than other mammals (Horn & de la Vega, 2016), while chiropterans (bats) showed higher %N, perhaps from investment in flight muscles (Studier et al., 1994). However, the low %N of passerine birds directly contradicts the pattern seen in chiropterans. Nevertheless, shorebirds (Order Charadriiformes), had a much higher %N, perhaps suggesting our passerine bird data are biased. Likely, we need more data to understand %N in passerine birds, as most measurements came from Sturges et al. (1974) (Table 4).

**TABLE 4:** Number of data points for each order

Order	%P points	%N points	N:P points
Anura	29 (15)	28 (13)	23 (11)
Urodela	37 (10)	31 (11)	28 (9)
Anseriformes	0	1 (1)	0
Charadriiformes	0	5 (1)	0
Galliformes	1 (1)	2 (2)	1 (1)
Passeriformes	16 (2)	12 (3)	10 (1)
Artiodactyla	4 (1)	0	0
Carnivora	0	6 (3)	0
Chiroptera	1 (1)	19 (2)	1 (1)
Eulipotyphla	1 (1)	2 (2)	1 (1)
Lagomorpha	1 (1)	1 (1)	1 (1)
Rodentia	19 (4)	26 (6)	19 (4)
Squamata	10 (4)	12 (4)	9 (3)
Testudines	5 (2)	6 (3)	5 (2)

*Note*: The number of sources is shown in parentheses.

### 4.3 Life stage

Despite evidence that life stage impacts organismal stoichiometry (e.g. Boros, Sály, et al., 2015; Pilati & Vanni, 2007; Stephens et al., 2017), it is often absent from large‐scale analyses of organismal stoichiometry (Andrieux et al., 2021; McIntyre & Flecker, 2010). However, %P changes with vertebrate development likely contrast what is found in other taxa. Because growth rate is highest in younger organisms, developing vertebrates will produce copious rRNA that results in a high RNA:DNA that inturn indicates both high growth rate and high %P (Buckley, 1984; Buckley et al., 2008; Elser et al., 2003). However, although growth rate decreases with age, likely reducing RNA:DNA, developing vertebrates may still showed dramatical increase in %P because of age‐dependent bone mineralization. Not only does the bone develop and mineralize with age, but the proportion of supportive bone should increase allometrically (Anderson et al., 1979) (note: in our dataset, life stage explains the stoichiometric variation with size; see Figures S15–S23).

Based on this study, we can conclude that bone development likely overrides the effect of decreasing RNA:DNA with age, as %P increased along ontogenetic trajectories. This pattern was most pronounced in amphibians we had few sub‐adult measurements from amniotes. The stability of %N suggests that increased %P rather than decreased %N drives N:P differences (Pilati & Vanni, 2007; Stephens et al., 2017 found the same pattern). Because proteins and nucleic acids have high %N and are always required (Sterner & Elser, 2002), N requirements are less fluid than P requirements. Additionally, because the collagen matrix of bone is N‐rich (Olszta et al., 2007), bone formation does not decrease %N but instead decreases N:P because of bone's P‐richness (Sterner & Elser, 2002).

### 4.4 Diet and habitat

**FIGURE 11:**
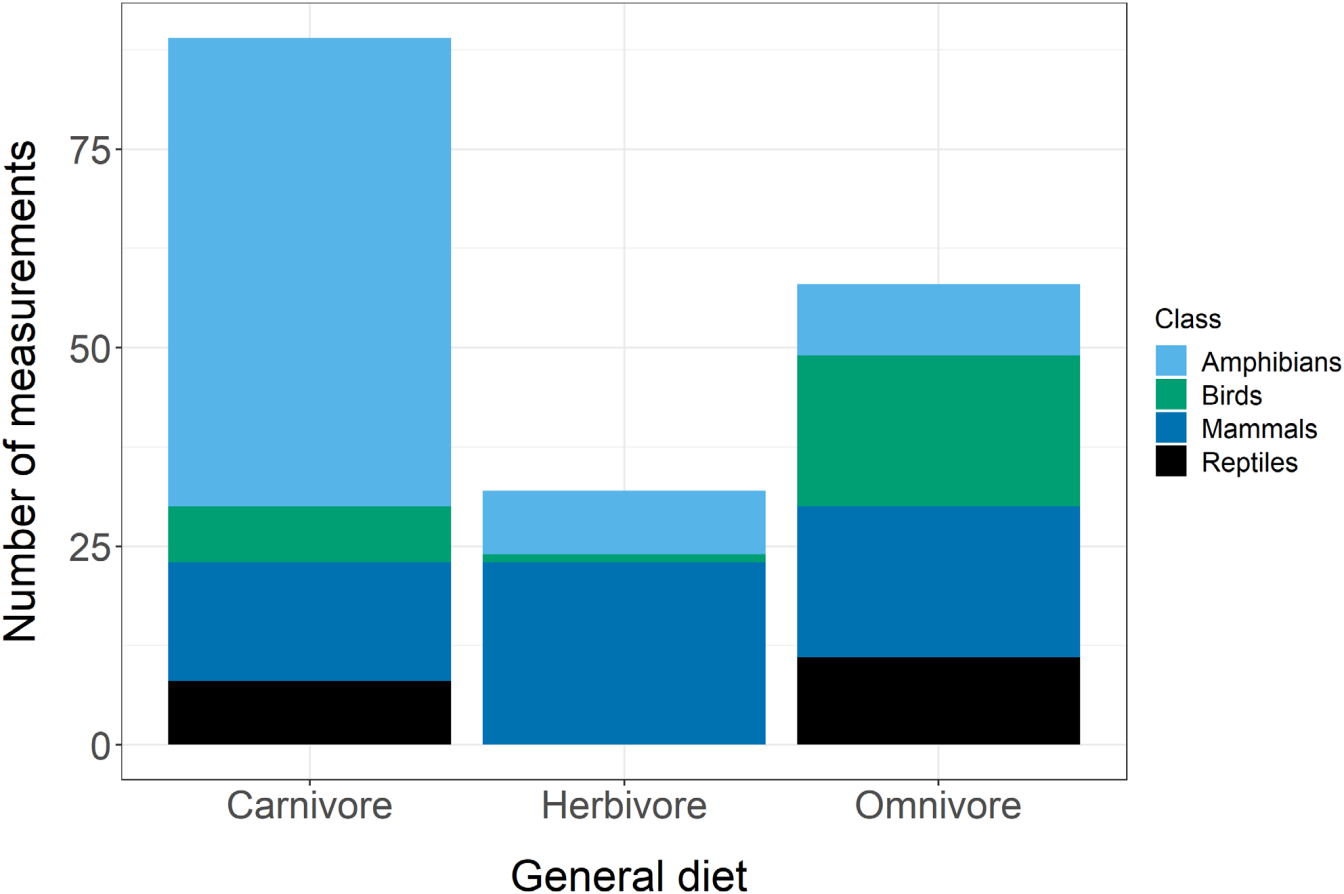
Taxonomic biases in diet distribution in our dataset.

Although diet explained some variation in %P models, relationships between diet and %P were inconsistent. This suggests that many vertebrates have evolved mechanisms to acquire sufficient nutrients from nutritionally poor substrates rather than evolving lowered body %P (Jeyasingh et al., 2014). Studies on fish (e.g. McIntyre & Flecker, 2010) also suggest that diet minimally impacts body stoichiometry. For example, some herbivorous catfish, although feeding on P‐poor foods, are heavily armored and thus maintain high %P (Hood et al., 2005). Other vertebrates show ontogenetic diet changes (Boros, Sály, et al., 2015; Bouchard & Bjorndal, 2006; Sterrett et al., 2015), in which adults switch from a nutritionally rich food to a nutritionally poor food, indicating that adult vertebrates have lower nutritional demand than juveniles. Decreased growth rates (Sterrett et al., 2015) and bone resorption cycles (Benstead et al., 2014) may allow adults to decrease their nutrient demand. Our results contrast Fagan et al. (2002) and Evans‐White et al. (2005), who suggested that carnivorous invertebrates may show the highest nutrient content. Nonetheless, we had strong taxonomic bias in dietary data, which may have contributed to our results (Figure 11). In general, however, diet is one of the least accurate traits reported in meta‐analyses because it can be reported in different ways and is subject to individual interpretation if specific data are unavailable. Furthermore, grouping animals into categories or trophic guilds may not accurately reflect their dietary stoichiometry or nutrient deficiencies (Vanni & McIntyre, 2016). As such, we hope our meta‐analysis encourages researchers to collect more specific dietary stoichiometry data that will better reflect organismal nutritional status.

We expected habitat to influence %P and N:P, as terrestrial animals theoretically require stronger bones than aquatic animals (Anderson et al., 1979; McIntyre & Flecker, 2010), while aquatic animals show a diverse range of bone mineral densities (Stein, 1989). Aquatic and terrestrial animals did not obviously differ in our dataset; furthermore, our vertebrates did not have higher %P than fishes (Table 3). However, taxonomic biases may have contributed to our results. For example, while aquatic testudines are higher in %P than other aquatic animals, they may be lower in %P than their terrestrial counterparts (or vice versa). Additionally, investment in non‐supportive bone influences the relationship between habitat and %P. For example, many fishes have non‐supportive bony structures that nonetheless give them high %P (Durston & El‐Sabaawi, 2017; Hood et al., 2005), and marine mammals that maintain neutral buoyancy have exceptionally dense bones (de Buffrénil et al., 2010).

### 4.5 Future directions

Our study shows that vertebrates are nutrient‐rich and may serve as high concentration P stores in diverse ecosystems. This combined with their long lives make them valuable nutrient stores (Lovich et al., 2018; Stanford et al., 2020). Many vertebrate species are currently endangered or threatened (e.g. Dirzo et al., 2014; Hoffmann et al., 2010; Moritz & Agudo, 2013). Removing vertebrates or reducing their numbers will fundamentally change nutrient cycles (Lovich et al., 2018; Wenger et al., 2019).

While we found taxonomic and life stage patterns, current biases in the literature may have enhanced or dampened relationships. While we had 39 unique amphibian species, we found relatively few measurements from other classes. Many orders were represented by a single species, and most measurements for some taxa (e.g. Order Passeriformes) came from a single source (Table 4). Additionally, most studies came from temperate areas despite the diversity of tropical vertebrates. Thus, we could not test how diverse biomes and latitudes affect stoichiometry. Further research should target these underrepresented species and geographic areas.

Future research should also focus on intraspecific characteristics that affect stoichiometry. For example, sex strongly affects bone mineral density and mineral storage in vertebrates. Hormones like estrogen regulate bone structure and function in females, allowing storage of minerals required for processes like egg formation, pregnancy, and lactation (Baker, 2013; Nilsson et al., 2001; Squire et al., 2017). Female birds even have medullary bone, which specifically stores minerals for eggshell production (Dacke et al., 1993; Squire et al., 2017). Some males also have sex‐specific bony traits. For example, male cervids often develop seasonal antlers (Moen et al., 1999; Price et al., 2005; Price & Allen, 2004). Sex will, therefore, affect elemental content differently in each taxon.

Additionally, since bone is central to vertebrate stoichiometry, future research should consider bone characteristics (e.g. using bone %P to measure bone mineral density may be valuable; Durston & El‐Sabaawi, 2017). Our knowledge of how bone affects stoichiometry and elemental demand is incomplete. Bone, unique among tissues, has a self‐destroying mechanism that allows vertebrates to draw upon mineral reserves sequestered in bone (Kular et al., 2012; Pasteris et al., 2008). This may cause vertebrate N:P to fluctuate over short periods, similar to how plants vary due to nutrient storage in vacuoles (Sterner & Elser, 2002). Bone also increases organismal calcium and magnesium (Neuman & Neuman, 1953; Pasteris et al., 2008) and so must also be considered when analyzing rarer elements. Incorporating bone physiology into studies will, therefore, further reveal how vertebrates fit into ecological stoichiometry.

## AUTHOR CONTRIBUTIONS

**Emily M. May:** Conceptualization (lead); data curation (lead); formal analysis (equal); investigation (lead); methodology (lead); project administration (supporting); validation (equal); visualization (lead); writing – original draft (lead); writing – review and editing (equal). **Rana W. El‐Sabaawi:** Conceptualization (supporting); data curation (supporting); formal analysis (equal); funding acquisition (lead); investigation (supporting); methodology (supporting); project administration (lead); supervision (lead); validation (equal); visualization (supporting); writing – original draft (supporting); writing – review and editing (equal).

## CONFLICT OF INTEREST

We declare no conflicts of interest.

## Supporting information


Appendix S1

Appendix S2

## Data Availability

Data are available from the Dryad Digital Repository: https://doi.org/10.5061/dryad.gqnk98spt.
