# Supplementary material for: Life stage and taxonomy the most important factors determining vertebrate stoichiometry: A meta‐analysis
Source: Ecol Evol. 2022 Oct 1;12(10):e9354. doi: 10.1002/ece3.9354 (PMC9526032; doi:10.1002/ece3.9354)
Supplement: Supplementary file 1 — Appendix S1 [file ECE3-12-e9354-s002.docx]

# Appendix S1: Supporting information

## Systematic review

### List of Web of Science categories used in systematic review

➢ Ecology

➢ Environmental sciences

➢ Marine and freshwater biology

➢ Evolutionary biology

➢ Zoology

➢ Limnology

➢ Biology

➢ Developmental biology

➢ Reproductive biology

➢ Ornithology

### Methodological comparison to Andrieux et al. (2021)

Table S1: Comparison of the methods used by Andrieux et al. (2021) and the methods used in our study.

| **Aspect** | **Andrieux et al. (2021) – for vertebrates** | **Our study** | |
| --- | --- | --- | --- |
| **Body parts examined** | -Whole-body measurements  -Partial-body measurements | | -Whole-body measurements |
| **Data format** | -Measurements on individuals  -Means, if individual measures unavailable | | -Means only (weighted) |
| **Factors evaluated** | -Taxonomic group  -Size  -Habitat  -Diet | | -Taxonomic group  -Life stage  -Size  -Habitat  -Diet |

### Carbon data


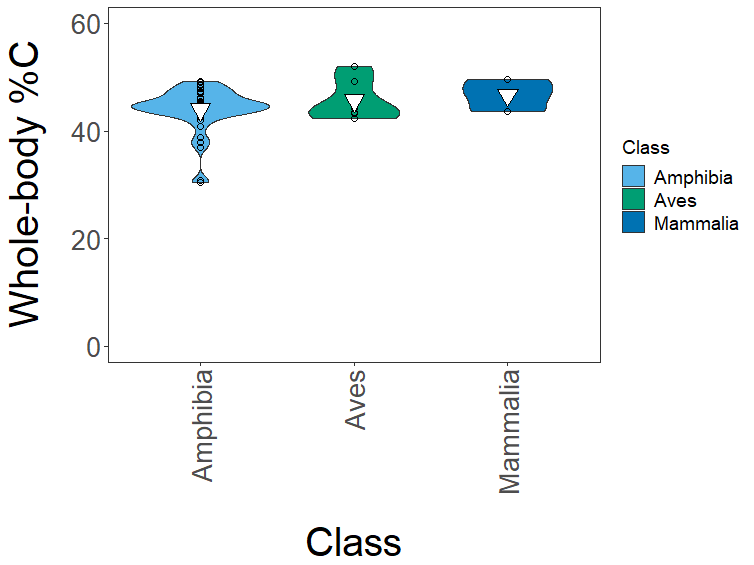


**Figure S1**: Relationship between %C and class found in our entire dataset. Inverted triangles show means.


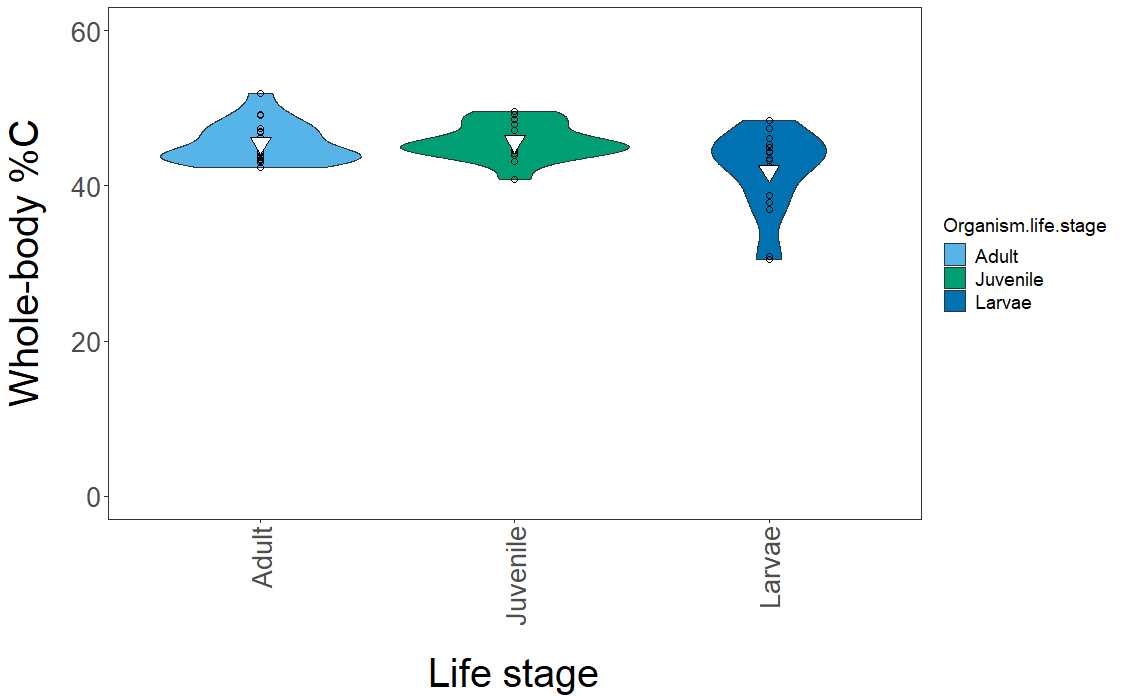


Figure S2: Relationship between %C and life stage found in our entire dataset. Inverted triangles show means.

### Detailed descriptions of variables (meta-data)

Table S2: General information extracted from each study in the meta-analysis.

| **Information** | **Description** | **Data extraction spreadsheet heading** |
| --- | --- | --- |
| **Accession number** | The unique ID of the source study. | ACC# |
| **Population number** | The ID of a group of individuals within a single source study. | Population |
| **Missing data** | Will indicate whether the authors should be contacted to supply further data. | Contact |
| **Animal evisceration** | Will indicate whether the animal was or was not eviscerated before its elemental content was determined (Yes or No). | Evisceration? |
| **Comments** | Any additional important information taken from the study in question. | Comments |

Table S3: Response variables extracted from each study (where possible).

| **Effect size** | **Description** | **Data extraction spreadsheet abbreviation** |
| --- | --- | --- |
| **% nitrogen in body** | The percentage of nitrogen (N) in the whole body of the organism | %N |
| **% phosphorus in body** | The percentage of phosphorus (P) in the whole body of the organism | %P |
| **Body N:P** | The ratio of N to P in the whole body of the organism (if applicable). Converted into a molar ratio. | N:P |
| **Body C:P (optional)** | The ratio of carbon (C) to P in the whole body of the organism. Converted into a molar ratio. | C:P |
| **Body C:N**  **(optional)** | The ratio of C to N in the whole body of the organism. Converted into a molar ratio. | C:N |
| **Body C:N:P**  **(optional)** | The C:N:P ratio of the whole body of the organism (optional). Converted into a molar ratio. | C:N:P |

Table S4: Factors extracted from each study (where possible).

| **Factor** | **Description** | **Data extraction spreadsheet heading** |
| --- | --- | --- |
| **Species** | The species of the organism studied (recorded as binomial scientific name) | Species |
| **Genus** | The genus of the organism studied | Genus |
| **Family** | The family of the organism studied | Family |
| **Order** | The order of the organism studied | Order |
| **Class** | The class of the organism studied | Class |
| **Sex** | If only one sex of organisms is under study, record as “M” or “F”. Only if applicable. | Sex |
| **Life stage** | The life stage of the organism, recorded as “larva” (in amphibians), “neonate” (in amniotes), “juvenile”, or “adult” | Life stage |
| **Habitat** | The general habitat of the organism, recorded as “marine”, “freshwater”, or “terrestrial” | Habitat |
| **Study location** | The location of the study (if available) | Location |
| **Diet (general)** | Whether the organism is “herbivorous”, “omnivorous” or “carnivorous”. If it differs by life stage, this will be recorded as the value at the life stage under study. | General diet |
| **Field or laboratory study** | Whether the organisms under study are “wild caught” or “lab reared” | Field or lab |

Table S5: Covariates extracted from each study (where possible).

| **Covariate** | **Description** | **Data extraction spreadsheet heading** |
| --- | --- | --- |
| **Temperature** | The temperature at the study location (in degrees Celsius) | Temp |
| **Mean dry mass** | The mean dry mass of individuals under study (in grams). | Dry mass |
| **Mean fresh mass** | The mean wet mass of individuals under study (in grams). | Fresh mass |

### Calculations used for weighting studies: %N and %P

We used a measure of weight that included a measure of study variance (Vi) and a measure of between-study variance (τ^2^), on the assumption that the true effect size (%N or %P of vertebrates) varied non-randomly. The following shows how we calculated these weights W_k_ for study k.


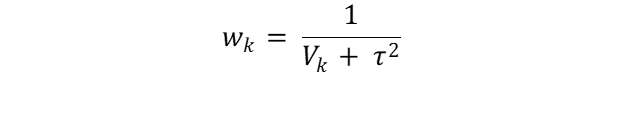


Calculating τ^2^ :

τ^2^ = Q-df/C, where:

Q = total variance

W_i_ = 1/V_i_

T_i_ = effect size estimate from study i

T. = effect size mean

df = degrees of freedom (k-1)

Q = ∑ W_i_ * (T_i_ – T.)^2^

T. = ∑ W_i_T_i_ / ∑ W_i_

C = ∑ W_i_ – (∑ W_i_^2^/∑ W_i_)

### Calculations used for weighting studies: N:P

*
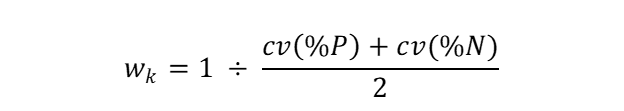
*

## Supplemental data figures


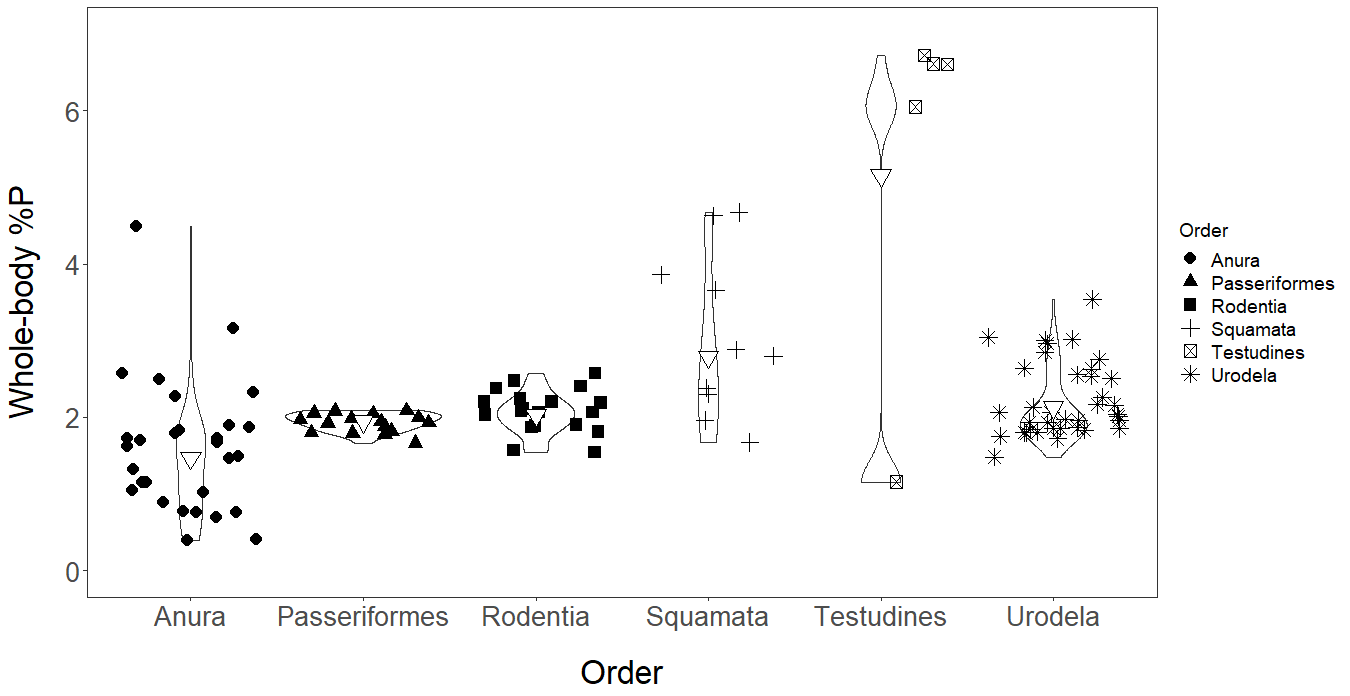


Figure S3: Relationship between whole-body %P and taxonomic order in vertebrates (only orders with n>2 are shown). Inverted triangles show means, and points are jittered. Different symbols used for each order to help distinguish jittered points.


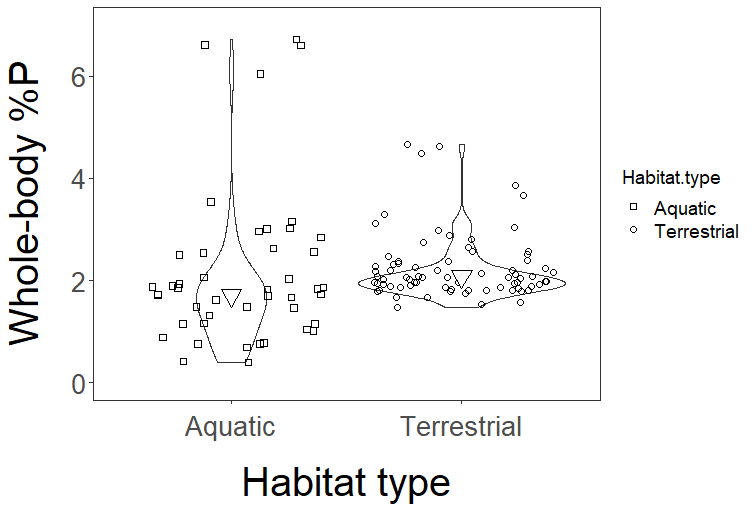


Figure S4: Relationships between habitat and whole-body %P. Inverted triangles show means, and points are jittered. Different symbols used for each habitat to help distinguish jittered points.


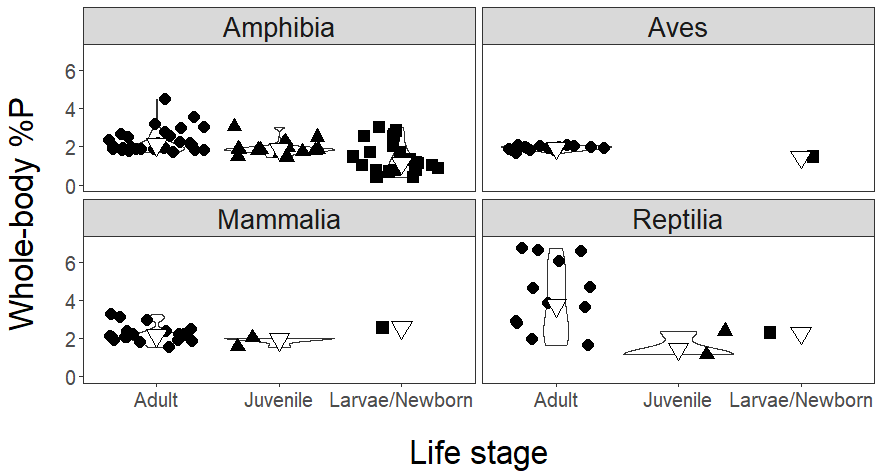


Figure S5: Relationships between life stage and whole-body %P, separated by class. Inverted triangles show means, and points are jittered. Different symbols used for each life stage to help distinguish jittered points.


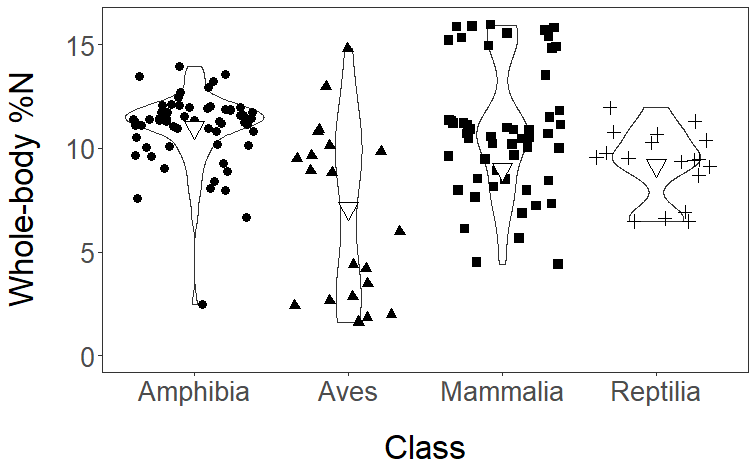


Figure S6: Relationship between whole-body %N and taxonomic class in vertebrates. Inverted triangles show means, and points are jittered. Different symbols used for each class to help distinguish jittered points.


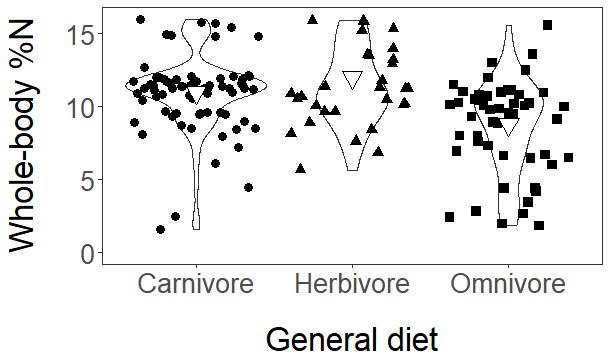


Figure S7: Relationships between diet and whole-body %N. Inverted triangles show means, and points are jittered. Different symbols used for each diet to help distinguish jittered points.


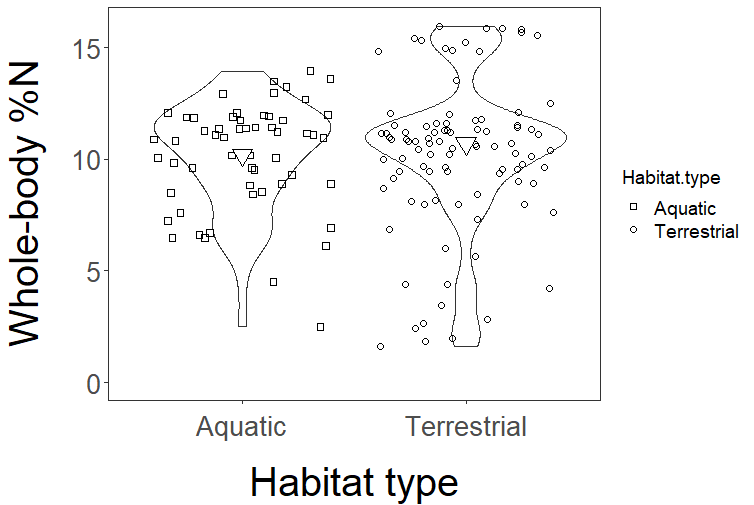


Figure S8: Relationships between habitat and whole-body %N. Inverted triangles show means, and points are jittered. Different symbols used for each habitat to help distinguish jittered points.


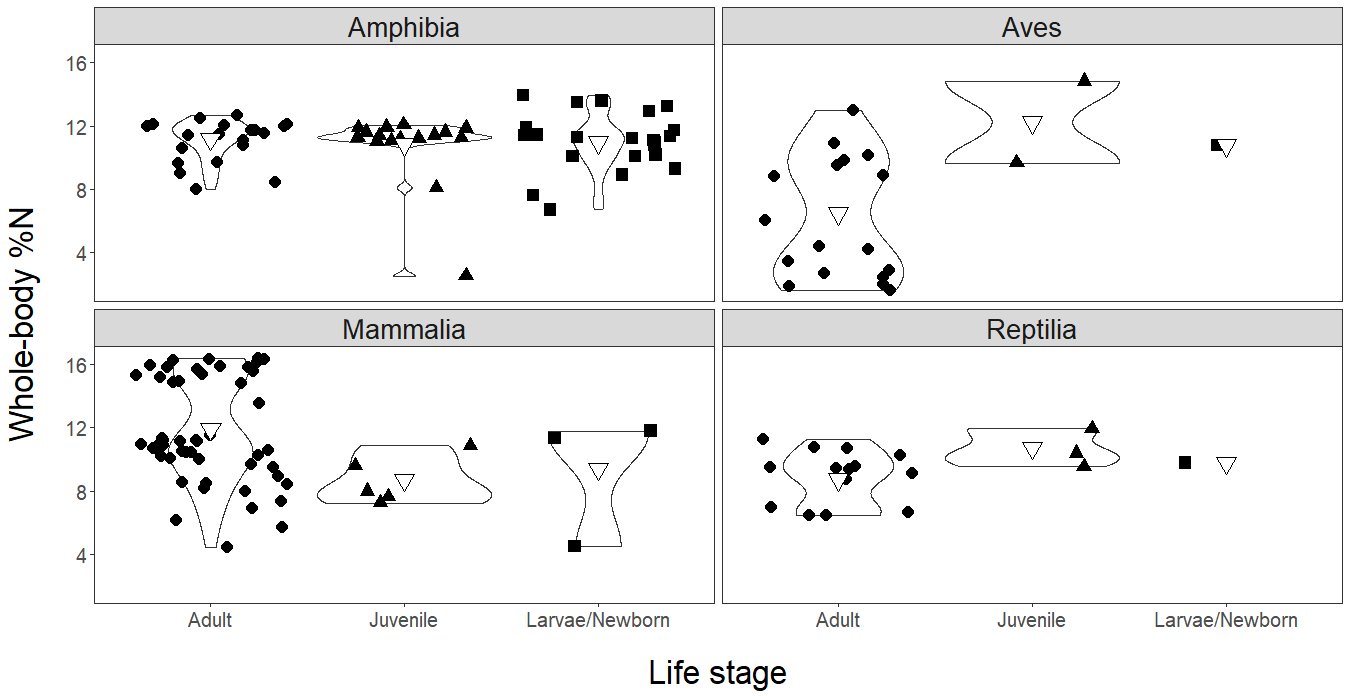
**Figure S9**: Relationships between life stage and whole-body %N, separated by class. Inverted triangles show means, and points are jittered. Different symbols used for each life stage to help distinguish jittered points.


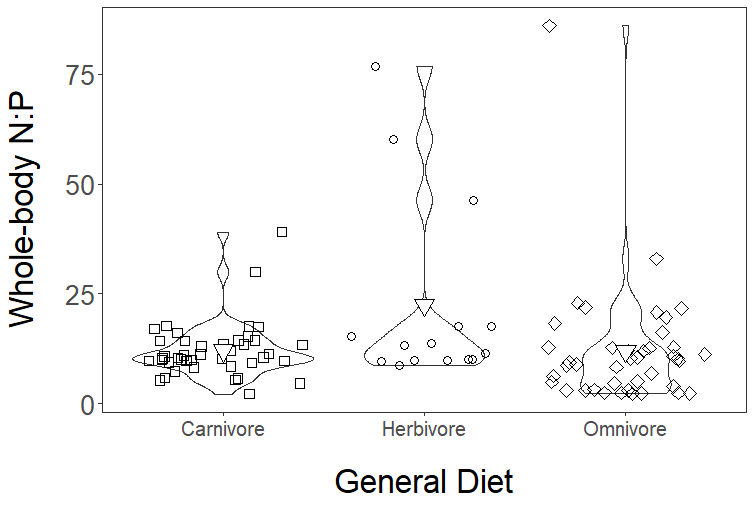


Figure S10: Relationship between diet and whole-body N:P. Inverted triangles show means, and points are jittered. Different symbols used for each diet to help distinguish jittered points.


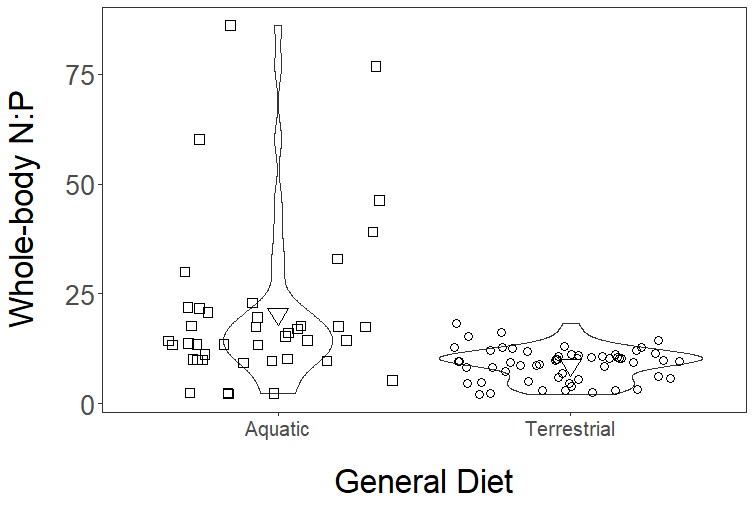
**Figure S11:** Relationships between habitat and whole-body N:P. Inverted triangles show means, and points are jittered. Different symbols used for each habitat to help distinguish jittered points.

## Model output tables

Table S6: Top four weighted general linear models used to describe whole-body %P in our whole dataset. Models ranked by ΔAICc.

| **Model name** | **Model formula** | **ΔAICc** | | **Model weight** |
| --- | --- | --- | --- | --- |
| **1** | **%P ~ class + life stage + diet** | **0** | **0.407** | |
| 2 | %P ~ class + life stage + diet + habitat | 2.06 | 0.145 | |
| 3 | %P ~ order + life stage + diet | 2.76 | 0.102 | |
| 4 | %P ~ class + order + life stage + diet | 2.76 | 0.102 | |

Table S7: Calculated estimates from best fit model for %P in our entire dataset (%P ~ class + life stage + general diet).

| **Variable** | **Estimate** | **Standard Error** | **P value** |
| --- | --- | --- | --- |
| **Intercept** | 0.81455 | 0.05772 | <2.0 X 10^-16^ |
| **Class(Aves)** | 0.15053 | 0.12552 | 0.232945 |
| **Class(Mammalia)** | 0.35455 | 0.12630 | 0.0058895 |
| **Class(Reptilia)** | 0.70977 | 0.11732 | 1.96 X 10^-8^ |
| **Life stage (Juvenile)** | -0.22007 | 0.09145 | 0.017745 |
| **Life stage (Larvae/neonate)** | -0.30442 | 0.10579 | 0.004798 |
| **General diet (Herbivore)** | -0.35721 | 0.13076 | 0.007322 |
| **General diet (Omnivore)** | -0.37957 | 0.10023 | 0.000247 |

Table S8: Top six equivalent weighted general linear models used to describe whole-body %N in our whole dataset. Models ranked by ΔAICc. Models including class were not used in model averaging.

| **Model name** | **Model formula** | **ΔAICc** | **Model weight** |
| --- | --- | --- | --- |
| 1 | %N ~ order | 0 | 0.167 |
| 2 | %N ~ class + order | 0 | 0.167 |
| 3 | %N ~ order + life stage | 0.59 | 0.124 |
| 4 | %N ~ class + order + life stage | 0.59 | 0.124 |
| 5 | %N ~ order + life stage + habitat | 1.89 | 0.065 |
| 6 | %N ~ class + order + life stage + habitat | 1.89 | 0.065 |

Table S9: Calculated estimates from averaged best fit model for %N in entire dataset. Averaged best fit model from 1) %N ~ order (weight = 0.60), 2) %N ~ order + habitat + life stage (weight = 0.12), and 3) %N ~ order + life stage (weight = 0.27). Values shown are from the full averaged model.

| **Variable** | **Estimate** | **Standard Error** | **P value** |
| --- | --- | --- | --- |
| **Intercept** | 2.177909 | 0.2725294 | <2.0 X 10^-16^ |
| **Order (Anura)** | 0.129127 | 0.286903 | 0.65266 |
| **Order (Artiodacyla)** | -0.179387 | 0.311172 | 0.56758 |
| **Order(Carnivora)** | -0.226950 | 0.296736 | 0.44829 |
| **Order(Charadriiformes)** | -0.156846 | 0.298965 | 0.60299 |
| **Order (Chiroptera)** | 0.537790 | 0.282769 | 0.05935 |
| **Order (Eulipotyphla)** | -0.272920 | 0.336571 | 0.42144 |
| **Order (Galliformes)** | 0.090885 | 0.346229 | 0.79461 |
| **Order (Lagomorpha)** | 0.228323 | 0.387971 | 0.55960 |
| **Order (Passeriformes)** | -0.888413 | 0.287091 | 0.00215 |
| **Order (Rodentia)** | 0.110046 | 0.281405 | 0.69823 |
| **Order (Squamata)** | 0.094825 | 0.287606 | 0.74375 |
| **Order (Testudines)** | -0.145587 | 0.296002 | 0.62582 |
| **Order (Urodela)** | 0.188074 | 0.280570 | 0.50629 |
| **Life stage (Juvenile)** | 0.046888 | 0.072503 | 0.51917 |
| **Life stage (Larvae/neonate)** | 0.041182 | 0.070892 | 0.56291 |
| **Habitat (Terrestrial)** | 0.009682 | 0.039435 | 0.80702 |

Table S10: Calculated estimates from averaged best fit model for %N in entire dataset. Averaged best fit model from 1) %N ~ order (weight = 0.60), 2) %N ~ order + habitat + life stage (weight = 0.12), and 3) %N ~ order + life stage (weight = 0.27). Values shown are conditional averages from the averaged model.

| **Variable** | **Estimate** | **Standard Error** | **P value** |
| --- | --- | --- | --- |
| **Intercept** | 2.17791 | 0.27292 | <2.0 X 10^-16^ |
| **Order (Anura)** | 0.12913 | 0.28450 | 0.65266 |
| **Order (Artiodacyla)** | -0.17939 | 0.31117 | 0.56758 |
| **Order(Carnivora)** | -0.22695 | 0.29674 | 0.44829 |
| **Order(Charadriiformes)** | 0.15685 | 0.29896 | 0.60299 |
| **Order (Chiroptera)** | 0.53779 | 0.28277 | 0.05935 |
| **Order (Eulipotyphla)** | -0.27292 | 0.33657 | 0.42144 |
| **Order (Galliformes)** | 0.09088 | 0.34623 | 0.79461 |
| **Order (Lagomorpha)** | 0.22832 | 0.38797 | 0.55960 |
| **Order (Passeriformes)** | -0.88842 | 0.28709 | 0.00215 |
| **Order (Rodentia)** | 0.11005 | 0.28141 | 0.69823 |
| **Order (Squamata)** | 0.09482 | 0.28761 | 0.74375 |
| **Order (Testudines)** | -0.14559 | 0.29600 | 0.62582 |
| **Order (Urodela)** | 0.18807 | 0.28057 | 0.50629 |
| **Life stage (Juvenile)** | 0.11800 | 0.06956 | 0.09264 |
| **Life stage (Larvae/neonate)** | 0.10364 | 0.07858 | 0.19091 |
| **Habitat (Terrestrial)** | 0.07864 | 0.08490 | 0.35858 |

Table S11: Equivalent weighted general linear models used to describe whole-body N:P in our whole dataset. Models ranked by ΔAICc.

| **Model name** | **Model formula** | **ΔAICc** | **Model weight** |
| --- | --- | --- | --- |
| 1 | N:P ~ order + diet | 0 | 0.187 |
| 2 | N:P ~ class + order + diet | 0 | 0.187 |
| 3 | N:P ~ order + life stage + diet | 0.90 | 0.119 |
| 4 | N:P ~ class + order + life stage + diet | 0.90 | 0.119 |
| 5 | N:P ~ order + life stage | 1.63 | 0.083 |
| 6 | N:P ~ class + order + life stage | 1.63 | 0.083 |

Table S12: Calculated estimates from averaged best fit model for N:P in entire dataset. Averaged best model from 1) N:P ~ order + diet (weight = 0.24), 2) N:P ~ class + order + diet (weight = 0.24), 3) N:P ~ order + life stage + diet (weight = 0.15), 4) N:P ~ class + order + life stage + diet (weight = 0.15), 5) N:P ~ order + life stage (weight = 0.11), and 6) N:P ~ class + order + life stage (weight = 0.11). Values shown are from the full averaged model.

| **Variable** | **Estimate** | **Standard Error** | **P value** |
| --- | --- | --- | --- |
| **Intercept** | 2.60587 | 0.13453 | <2.0 X 10^-16^ |
| **Order (Chiroptera)** | -0.28714 | 0.48367 | 0.55543 |
| **Order (Eulipotyphla)** | -1.46667 | 0.46836 | 0.00186 |
| **Order (Galliformes)** | 0.55702 | 0.89603 | 0.53458 |
| **Order (Lagomorpha)** | -0.45523 | 0.57613 | 0.43355 |
| **Order (Passeriformes)** | -0.85830 | 0.87928 | 0.32921 |
| **Order (Rodentia)** | -0.33010 | 0.36011 | 0.35982 |
| **Order (Squamata)** | 0.06072 | 0.93088 | 0.94802 |
| **Order (Testudines)** | -0.90483 | 0.92549 | 0.32842 |
| **Order (Urodela)** | -0.17350 | 0.12922 | 0.18474 |
| **Diet (Herbivore)** | 0.32813 | 0.23214 | 0.16019 |
| **Diet (Omnivore)** | 0.33344 | 0.21978 | 0.13124 |
| **Class (Aves)** | -0.85830 | 0.87928 | 0.32921 |
| **Class (Mammalia)** | -0.33010 | 0.36011 | 0.35982 |
| **Class (Reptilia)** | -0.90483 | 0.92549 | 0.32842 |
| **Life stage (Juvenile)** | 0.07184 | 0.09325 | 0.44396 |
| **Life stage (Larvae/neonate)** | 0.14836 | 0.18143 | 0.41541 |

Table S13: Calculated estimates from averaged best fit model for N:P in entire dataset. Averaged best model from 1) N:P ~ order + diet (weight = 0.24), 2) N:P ~ class + order + diet (weight = 0.24), 3) N:P ~ order + life stage + diet (weight = 0.15), 4) N:P ~ class + order + life stage + diet (weight = 0.15), 5) N:P ~ order + life stage (weight = 0.11), and 6) N:P ~ class + order + life stage (weight = 0.11). Values shown are conditional averages from the averaged model.

| **Variable** | **Estimate** | **Standard Error** | **P value** |
| --- | --- | --- | --- |
| **Intercept** | 2.60587 | 0.13453 | < 2.0 X 10^-16^ |
| **Order (Chiroptera)** | -0.28714 | 0.48367 | 0.55543 |
| **Order (Eulipotyphla)** | -1.46667 | 0.46836 | 0.00186 |
| **Order (Galliformes)** | 0.55702 | 0.89603 | 0.53458 |
| **Order (Lagomorpha)** | -0.45523 | 0.57613 | 0.43355 |
| **Order (Passeriformes)** | -1.71660 | 0.27002 | < 2.0 X 10^-16^ |
| **Order (Rodentia)** | -0.66019 | 0.20354 | 0.00127 |
| **Order (Squamata)** | 0.06072 | 0.93088 | 0.94802 |
| **Order (Testudines)** | -1.80965 | 0.27501 | < 2.0 X 10^-16^ |
| **Order (Urodela)** | -0.17350 | 0.12922 | 0.18474 |
| **Diet (herbivore)** | 0.41671 | 0.17756 | 0.02064 |
| **Diet (omnivore)** | 0.42345 | 0.15242 | 0.00613 |
| **Class (Aves)** | -1.71660 | 0.27002 | < 2.0 X 10^-16^ |
| **Class (Mammalia)** | -0.66019 | 0.20354 | 0.00127 |
| **Class (Reptilia)** | -1.80965 | 0.27501 | < 2.0 X 10^-16^ |
| **Life stage (Juvenile)** | 0.13844 | 0.08681 | 0.11587 |
| **Life stage (Larvae/neonate)** | 0.28588 | 0.15528 | 0.06852 |

Table S14: Summary of the top four weighted general linear models used to describe whole-body %P in adult vertebrates. Models are ranked by ΔAICc.

| **Model name** | **Model formula** | **ΔAICc** | **Model weight** |
| --- | --- | --- | --- |
| 1 | %P ~ order +  diet | 0 | 0.197 |
| 2 | %P ~ class + order + diet | 0 | 0.197 |
| 3 | %P ~ order | 0.03 | 0.195 |
| 4 | %P ~ class + order | 0.03 | 0.195 |

Table S15: Calculated estimates from averaged best fit model for %P in adult-only dataset. Averaged best model from 1) %P ~ diet + order (weight = 0.21) and 3) %P ~ order (weight = 0.79). Values shown are from the full averaged model.

| **Variable** | **Estimate** | **Standard Error** | **P value** |
| --- | --- | --- | --- |
| **Intercept** | 0.941005 | 0.082762 | <2.0 X 10^-16^ |
| **Order (Chiroptera)** | 0.251431 | 0.218968 | 0.25958 |
| **Order (Eulipotyphla)** | 0.186313 | 0.223901 | 0.41391 |
| **Order (Lagomorpha)** | 0.104120 | 0.243782 | 0.67433 |
| **Order (Passeriformes)** | -0.296092 | 0.104213 | 0.00527 |
| **Order (Rodentia)** | -0.229748 | 0.113520 | 0.04657 |
| **Order (Squamata)** | 0.181012 | 0.110878 | 0.10896 |
| **Order (Testudines)** | 0.922727 | 0.138955 | < 2.0 X 10^-16^ |
| **Order (Urodela)** | -0.172740 | 0.092932 | 0.06800 |
| **Diet (Herbivore)** | 0.043436 | 0.107157 | 0.68747 |
| **Diet (Omnivore)** | 0.006468 | 0.046740 | 0.89183 |

Table S16: Calculated estimates from averaged best fit model for %P in adult-only dataset. Averaged best model from 1) %P ~ diet + order (weight = 0.21) and 3) %P ~ order (weight = 0.79). Values shown are conditional averages from the averaged model.

| **Variable** | **Estimate** | **Standard Error** | **P value** |
| --- | --- | --- | --- |
| **Intercept** | 0.94101 | 0.08276 | < 2.0 X 10^-16^ |
| **Order (Chiroptera)** | 0.25143 | 0.21897 | 0.25958 |
| **Order (Eulipotyphla)** | 0.18631 | 0.22390 | 0.41391 |
| **Order (Lagomorpha)** | 0.10412 | 0.24378 | 0.67433 |
| **Order (Passeriformes)** | -0.29609 | 0.10421 | 0.00527 |
| **Order (Rodentia)** | -0.22975 | 0.11352 | 0.04657 |
| **Order (Squamata)** | 0.18101 | 0.11088 | 0.10896 |
| **Order (Testudines)** | 0.92273 | 0.13896 | < 2.0 X 10^-16^ |
| **Order (Urodela)** | -0.17274 | 0.09293 | 0.06800 |
| **Diet (Herbivore)** | 0.20234 | 0.14607 | 0.17401 |
| **Diet (Omnivore)** | 0.03013 | 0.09728 | 0.76116 |

Table S17: Summary of the top four weighted general linear models used to describe whole-body %N in adult vertebrates. Models are ranked by ΔAICc.

| **Model name** | **Model formula** | **ΔAICc** | **Model weight** |
| --- | --- | --- | --- |
| 1 | %N ~ order | 0 | 0.347 |
| 2 | %N ~ class | 0 | 0.347 |
| 3 | %N ~ order + habitat | 2.75 | 0.088 |
| 4 | %N ~ class + order + habitat | 2.75 | 0.088 |

Table S18: Calculated estimates from best fit %N model for the adult-only dataset (%N ~ order).

| **Variable** | **Estimate** | **Standard Error** | **P value** |
| --- | --- | --- | --- |
| **Intercept** | 2.1779 | 0.2245 | 1.49 X 10^-15^ |
| **Order (Anura)** | 0.3140 | 0.2510 | 0.2143 |
| **Order (Artiodactyla)** | -0.3475 | 0.2750 | 0.2097 |
| **Order (Carnivora)** | -0.1461 | 0.2593 | 0.5746 |
| **Order (Charadriiformes)** | 0.1568 | 0.2460 | 0.5253 |
| **Order (Chiroptera)** | 0.5475 | 0.2304 | 0.0196 |
| **Order (Eulipotyphla)** | -0.2632 | 0.275 | 0.3411 |
| **Order (Lagomorpha)** | 0.2380 | 0.3175 | 0.4555 |
| **Order (Passeriformes)** | -1.0015 | 0.2345 | 4.92 X 10^-5^ |
| **Order (Rodentia)** | 0.1241 | 0.2294 | 0.5900 |
| **Order (Squamata)** | 0.1078 | 0.2355 | 0.6482 |
| **Order (Testudines)** | -0.2874 | 0.2510 | 0.2553 |
| **Order (Urodela)** | 0.1886 | 0.2314 | 0.4172 |

Table S19: Summary of the top four weighted general linear models used to describe whole-body N:P in adult vertebrates. Models are ranked by ΔAICc.

| **Model name** | **Model formula** | **ΔAICc** |  | **Model weight** |
| --- | --- | --- | --- | --- |
| 1 | N:P ~ order + diet | 0 |  | 0.315 |
| 2 | N:P ~ class + order + diet | 0 |  | 0.315 |
| 3 | N:P ~ order + diet + habitat | 1.42 |  | 0.155 |
| 4 | N:P ~ class + order + diet + habitat | 1.42 |  | 0.155 |

Table S20: Calculated estimates from averaged best fit model for N:P in adult-only dataset. Averaged best model from 1)N:P ~ order + diet (weight = 0.83) and 2)N:P ~ order + diet + habitat type (weight = 0.17). Values shown are from the full averaged model.

| **Variable** | **Estimate** | **Standard Error** | **P value** |
| --- | --- | --- | --- |
| **Intercept** | 1.521342 | 0.294960 | 5.0 X 10^-7^ |
| **Order (Chiroptera)** | 0.477030 | 0.379414 | 0.221487 |
| **Order (Eulipotyphla)** | -0.693430 | 0.347624 | 0.052415 |
| **Order (Lagmorpha)** | 0.439551 | 0.372048 | 0.250627 |
| **Order (Passeriformes)** | -0.587947 | 0.258253 | 0.026843 |
| **Order (Rodentia)** | 0.602337 | 0.256738 | 0.022527 |
| **Order (Squamata)** | 0.222939 | 0.276767 | 0.433462 |
| **Order (Testudines)** | -1.008418 | 0.281060 | 0.000484 |
| **Order (Urodela)** | 0.810778 | 0.297466 | 0.008039 |
| **Diet (Herbivore)** | 0.197523 | 0.201715 | 0.340999 |
| **Diet (Omnivore)** | 0.324373 | 0.152026 | 0.038007 |
| **Habitat (Terrestrial)** | -0.009741 | 0.058891 | 0.871813 |

Table S21: Calculated estimates from averaged best fit model for N:P in adult-only dataset. Averaged best model from 1)N:P ~ order + diet (weight = 0.83) and 2)N:P ~ order + diet + habitat type (weight = 0.17). Values shown are conditional averages from the averaged model.

| **Variable** | **Estimate** | **Standard Error** | **P value** |
| --- | --- | --- | --- |
| **Intercept** | 1.52134 | 0.29496 | 5.0 X 10^-7^ |
| **Order (Chiroptera)** | 0.47703 | 0.37941 | 0.221487 |
| **Order (Eulipotyphla)** | -0.69343 | 0.34762 | 0.052415 |
| **Order (Lagmorpha)** | 0.43955 | 0.37205 | 0.250627 |
| **Order (Passeriformes)** | -0.58795 | 0.25825 | 0.026843 |
| **Order (Rodentia)** | 0.60234 | 0.25674 | 0.022527 |
| **Order (Squamata)** | 0.22294 | 0.27677 | 0.433462 |
| **Order (Testudines)** | -1.00842 | 0.28106 | 0.000484 |
| **Order (Urodela)** | 0.81078 | 0.29747 | 0.008039 |
| **Diet (Herbivore)** | 0.19752 | 0.20172 | 0.340999 |
| **Diet (Omnivore)** | 0.32437 | 0.15203 | 0.038007 |
| **Habitat (Terrestrial)** | -0.05818 | 0.13378 | 0.672539 |

## Amphibian analysis

Note: We also included an “order*life stage” interaction term for our amphibian dataset; we did not include this term elsewhere because very few amniotes in our study were sub-adults.

***Phosphorus***

Amphibians had a mean %P of 1.76% (s^2^ = 0.41; n = 66). The model **%P ~ order + family + life stage + order*life stage** (**Table S23**) best explained variation (**Figures S12-S14**). Order Urodela showed higher %P (2.08%; s^2^ = 0.16; n = 37) than Order Anura (1.31%; s^2^ = 0.40; n = 29); however, life stage affected order relationships, as adult anurans (2.28%; s^2^ = 0.43; n = 6) had higher %P than adult urodelans (2.08%; s^2^ = 0.14; n = 23). Our main effect of life stage showed the same patterns as in our other datasets, with adults (2.11%; s^2^ = 0.18; n = 29) having higher %P than juveniles (1.88%; s^2^ = 0.11; n = 15) and larvae/neonates (1.16%; s^2^ = 0.44; n = 22). However, this was more pronounced in anurans, with urodelans having a dampened effect of life stage and higher %P in larvae/neonates than in adults (**Figure S12**). This may have been because all larval/neonate urodelans in our dataset were the same species (*Eurycea cirrigera*). Family Ambystomatidae (2.01%; s^2^ = 0.085; n = 7) and Family Salamandridae (3.17%; s^2^ = 0.050; n = 2) likely drove family relationships, as they had higher %P than other amphibian families (**Figure S13**). Most families had low sample sizes, potentially masking other relationships.

Table S22: Calculated estimates from best fit model for %P in the amphibian-only dataset (%P ~ order + family + life stage + order*life stage).

| **Variable** | **Estimate** | **Standard Error** | **P value** |
| --- | --- | --- | --- |
| **Intercept** | 0.9286 | 0.2653 | 0.000952 |
| **Order (Urodela)** | 0.3175 | 0.3196 | 0.325027 |
| **Life stage (Juvenile)** | -0.3445 | 0.1331 | 0.012433 |
| **Life stage (Larva/neonate)** | -1.0487 | 0.1277 | 5.19 X 10^-11^ |
| **Family (Ambystomatidae)** | -0.4863 | 0.1888 | 0.012802 |
| **Family (Bufonidae)** | -0.2009 | 0.2763 | 0.470311 |
| **Family (Dendrobatidae)** | -0.8158 | 0.3289 | 0.016330 |
| **Family (Hylidae)** | -0.2011 | 0.2579 | 0.439012 |
| **Family (Pipidae)** | 0.2207 | 0.3528 | 0.534368 |
| **Family (Plethodontidae)** | -0.5009 | 0.18292 | 0.008388 |
| **Family (Ranidae)** | 0.2284 | 0.2424 | 0.350460 |
| **Urodela*Juvenile** | 0.2283 | 0.1910 | 0.237389 |
| **Urodela*Larva** | 1.1729 | 0.1633 | 2.29 X 10^-9^ |


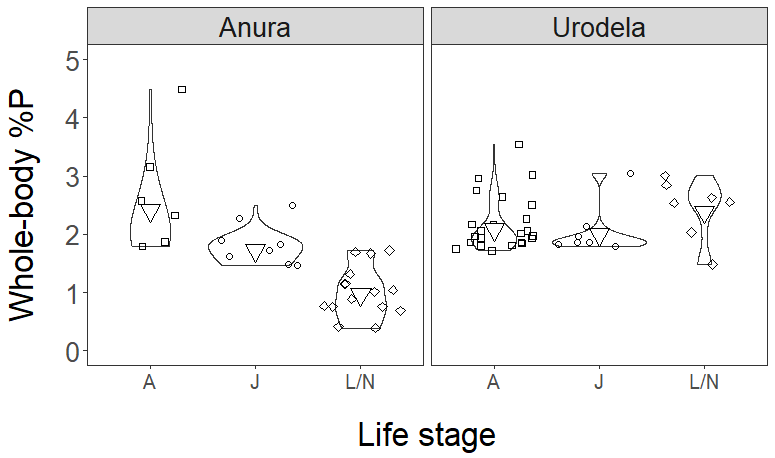
**Figure S12**: Interaction between order and life stage (A = adults, J = juveniles, L/N = larvae/neonates) in our amphibian dataset. Points have been jittered. Inverted triangles show means. Different symbols used for each life stage to distinguish jittered points.


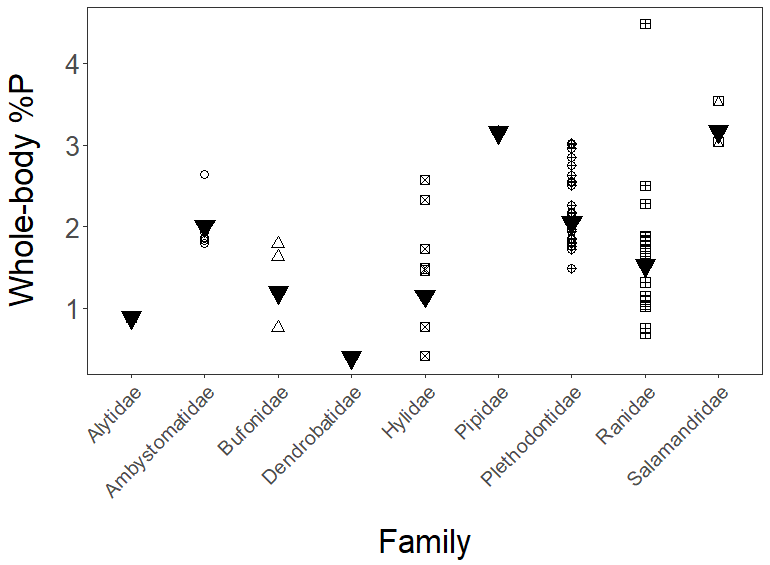


Figure S13: Relationship between %P and amphibian family. Black inverted triangles show weighted means. Family Alytidae, Family Dendrobatidae, and Family Pipidae have n = 1.

***Nitrogen***

The mean %N of all amphibians in our dataset was 10.99% (s^2^ = 3.45; n = 59). Tested variables explained little variation, and the best model was the null model. Order, the variable in our second-best model, had little noticeable influence; anurans (10.80%; s^2^ = 6.59; n = 28) and urodelans (11.17%: s^2^ = 1.32; n = 31) had similar %N (**Figure S14**). Relationship between family and %N shown in **Figure S15**.


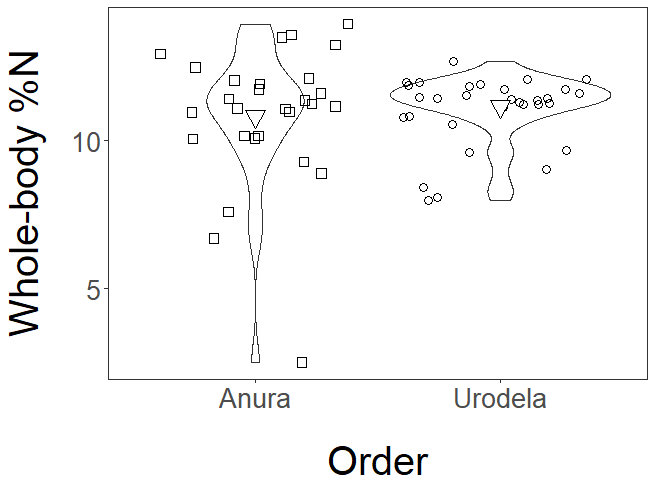


**Figure S14**: Whole-body %N in anurans and urodelans. Points have been jittered. Inverted triangles show weighted means.


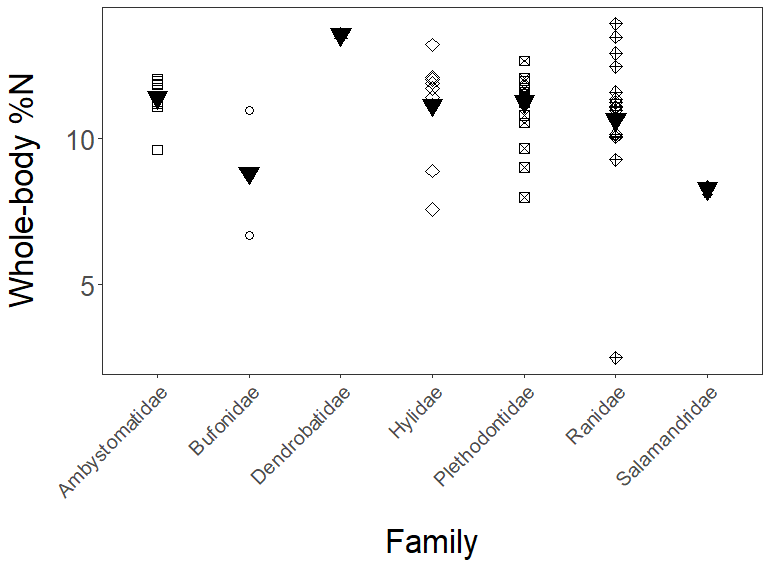


Figure S15: Relationship between %N and amphibian family. Black inverted triangles show weighted means. Family Dendrobatidae has n = 1.

## Visualizations of dry mass

The effect of dry mass on %P, %N, and N:P was either not evident or was influenced heavily by life stage. We have plotted the effects of dry mass on %P, %N, and N:P below, facetted by class, habitat, and life stage. All plots are transformed by log_e_ because of several very large organisms (e.g. phocids).


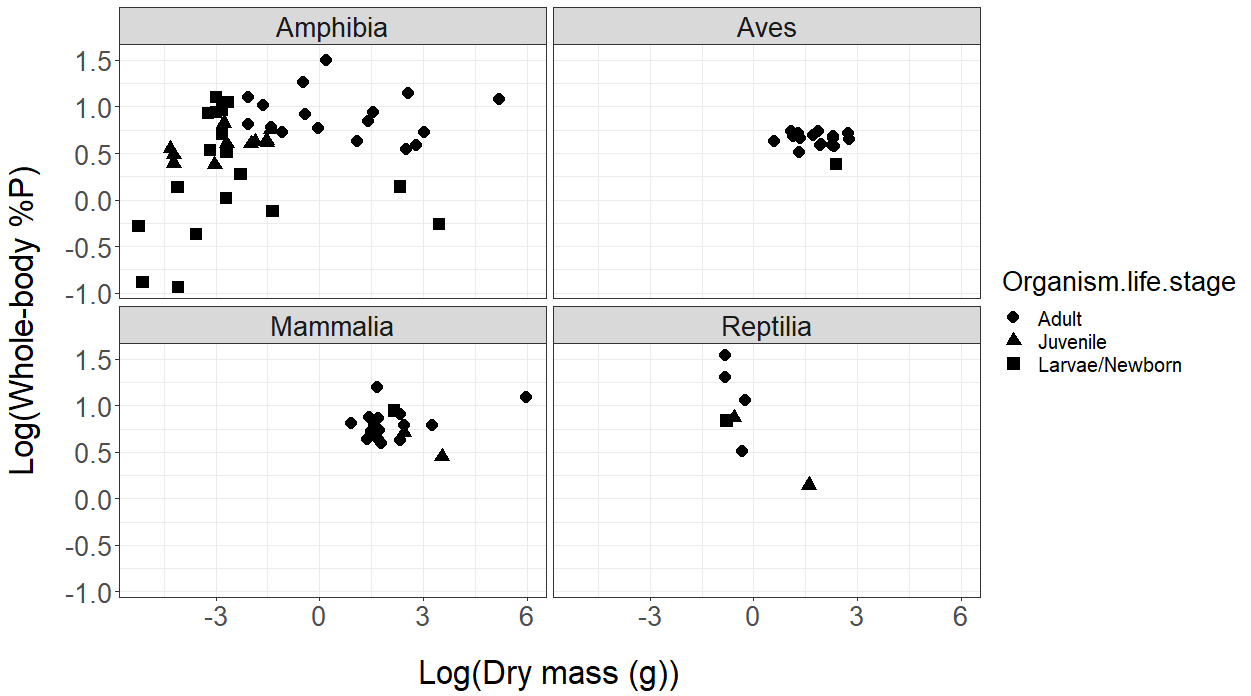


Figure S16: Effect of dry mass on whole-body %P (both transformed by log_e_), facetted by class. Different shapes indicate different life stages.


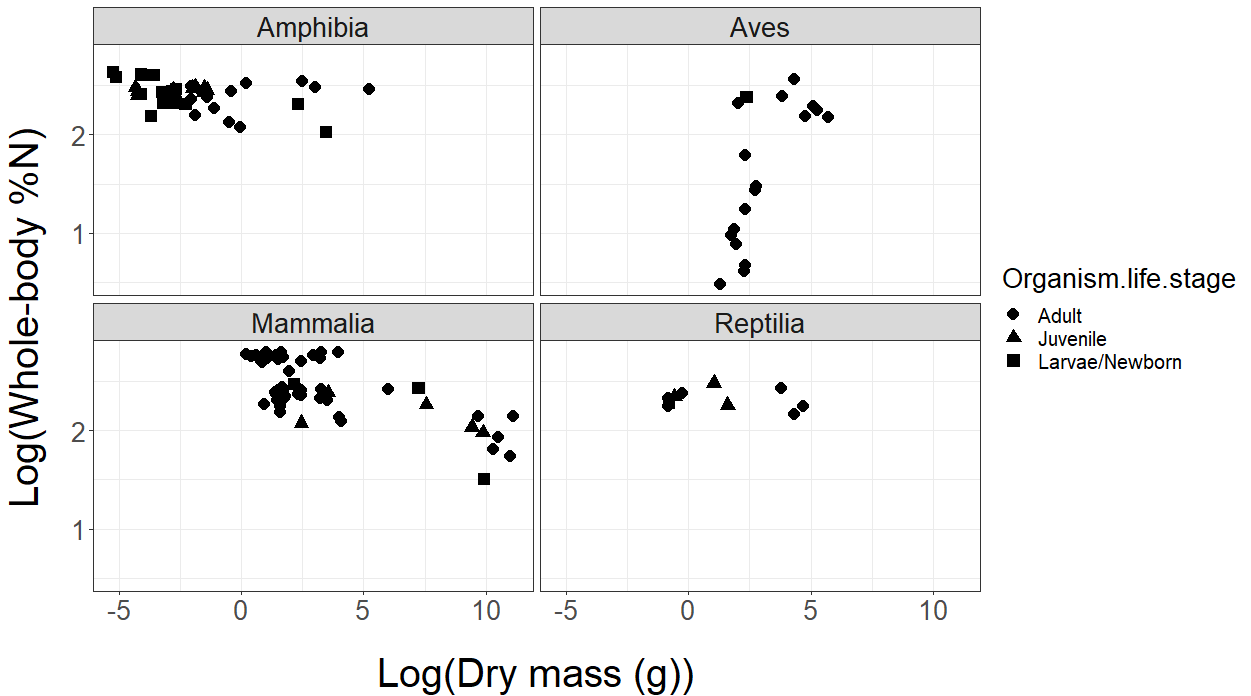


Figure S17: Effect of dry mass on whole-body %N (both transformed by log_e_), facetted by class. Different shapes indicate different life stages.


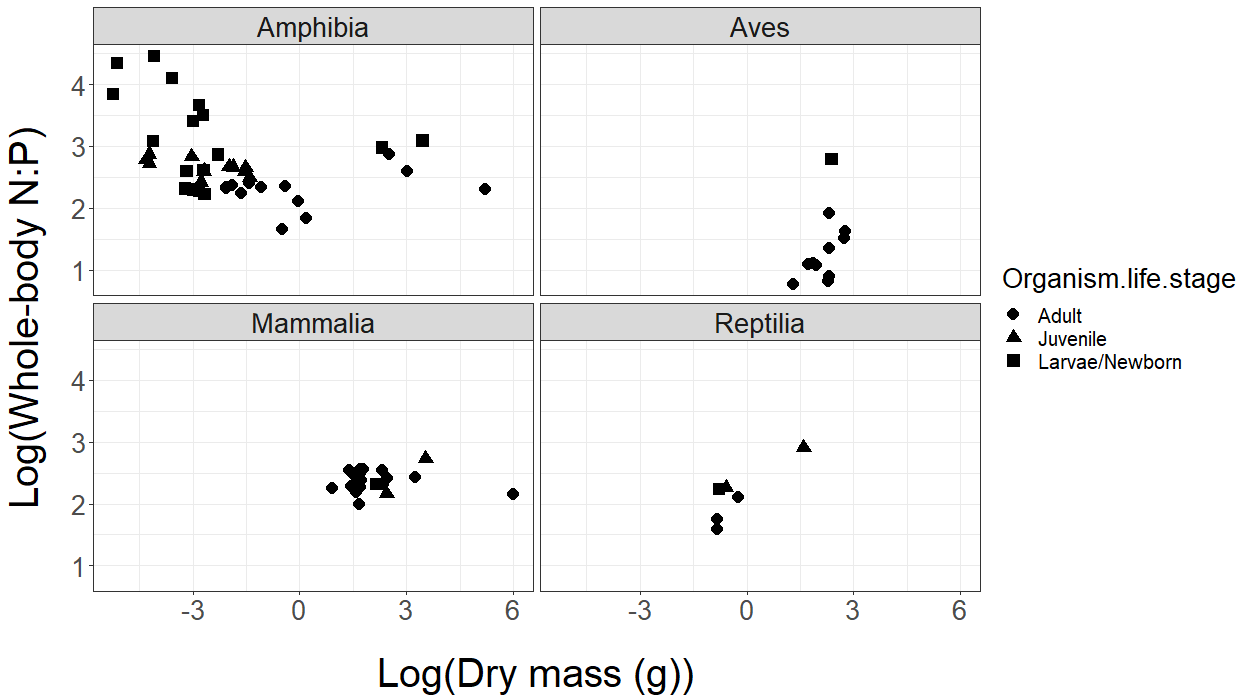


Figure S18: Effect of dry mass on whole-body N:P (both transformed by log_e_), facetted by class. Different shapes indicate different life stages.


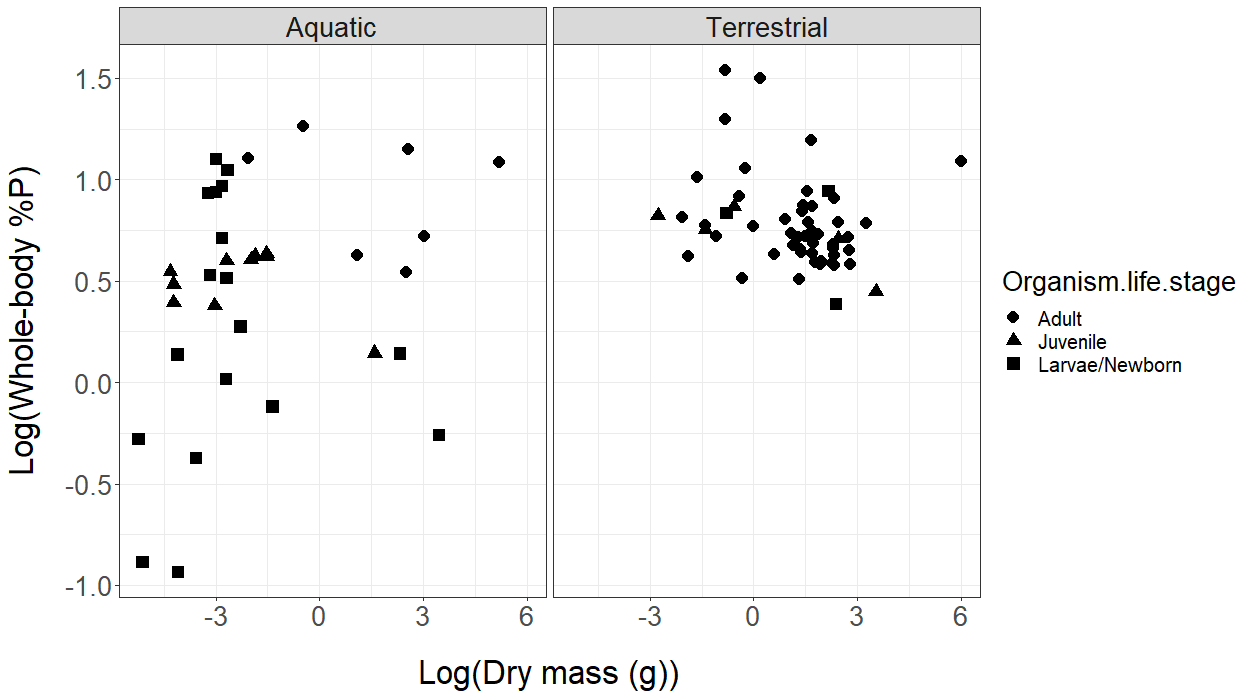


Figure S19: Effect of dry mass on whole-body %P (both transformed by log_e_), facetted by habitat type.


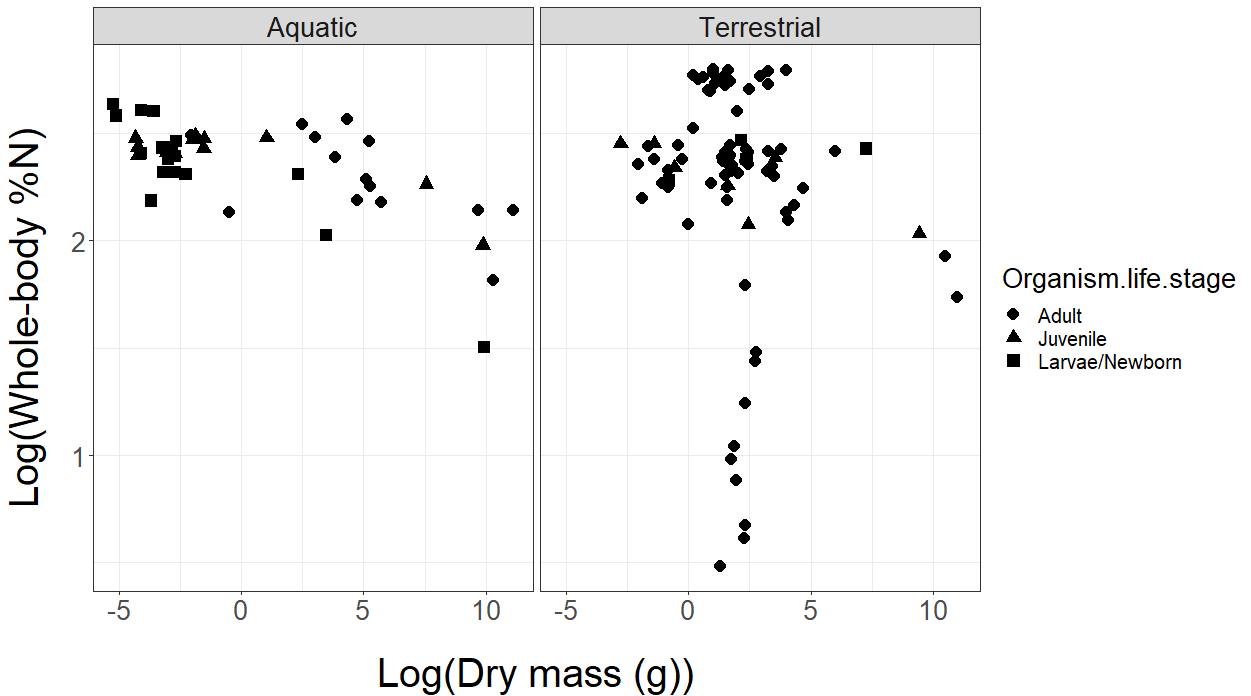


**Figure S20:** Effect of dry mass on whole-body %N (both transformed by log_e_), facetted by habitat type.


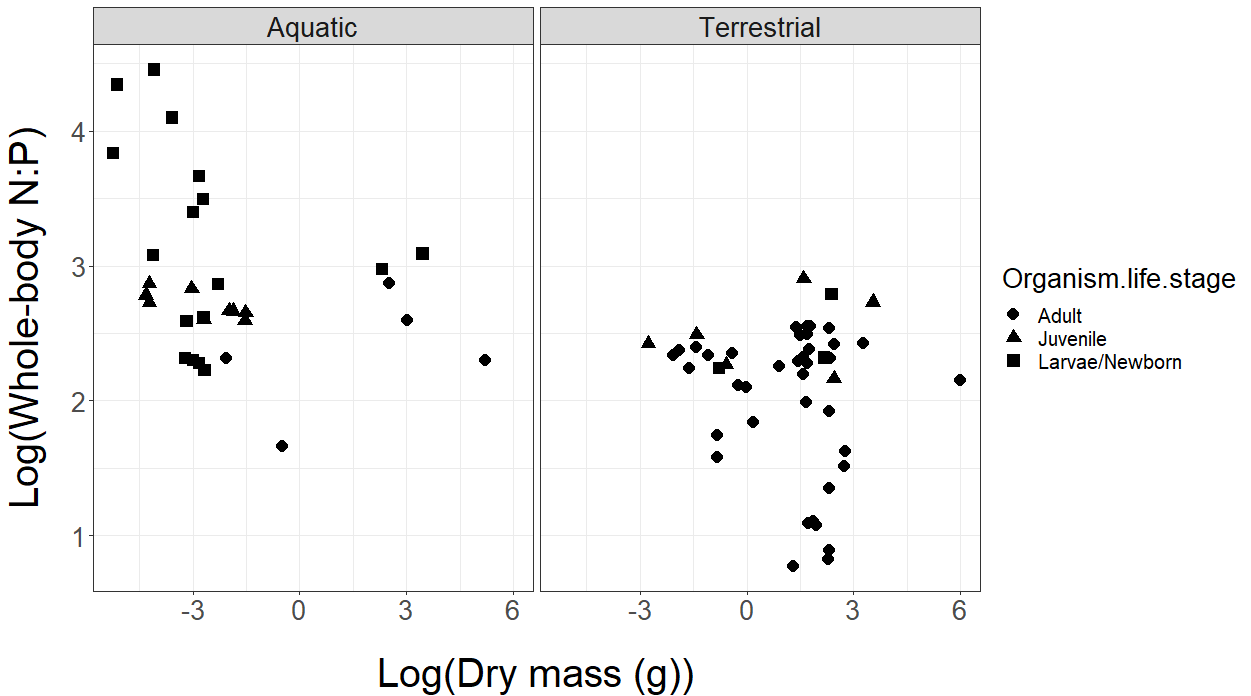


Figure S21: Effect of dry mass on whole-body N:P (both transformed by log_e_), facetted by habitat type.


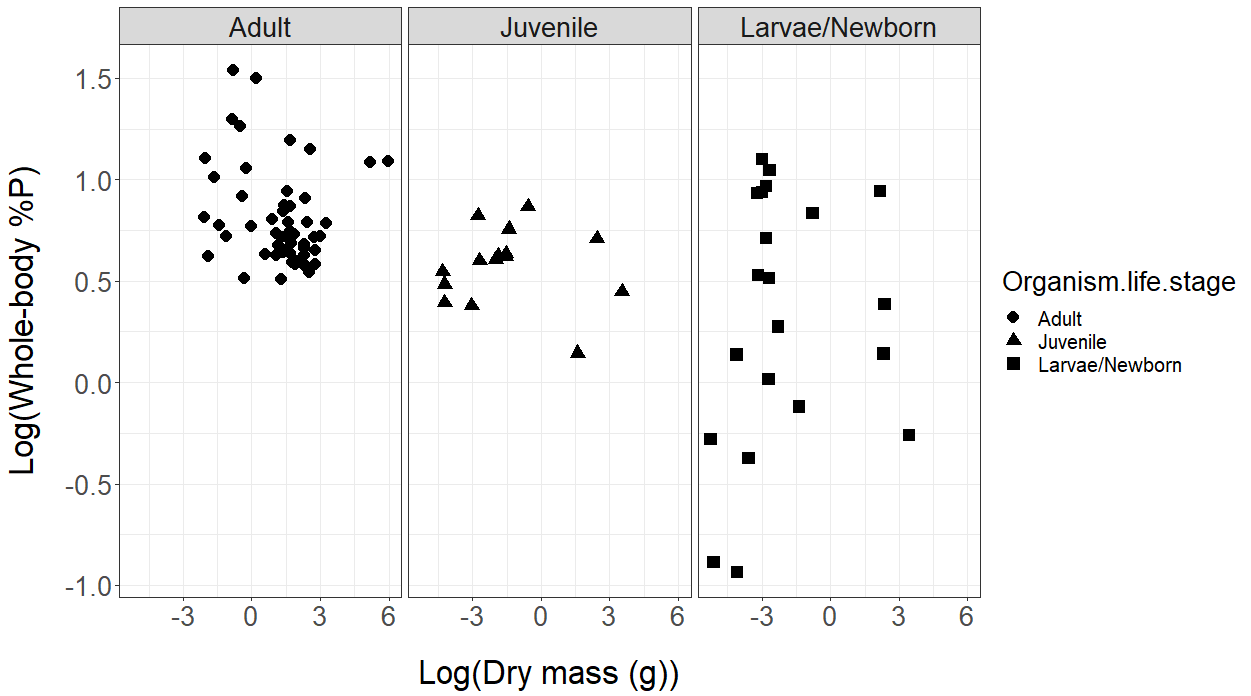


**Figure S22**: Effect of dry mass on whole-body %P (both transformed by log_e_), facetted by life stage.


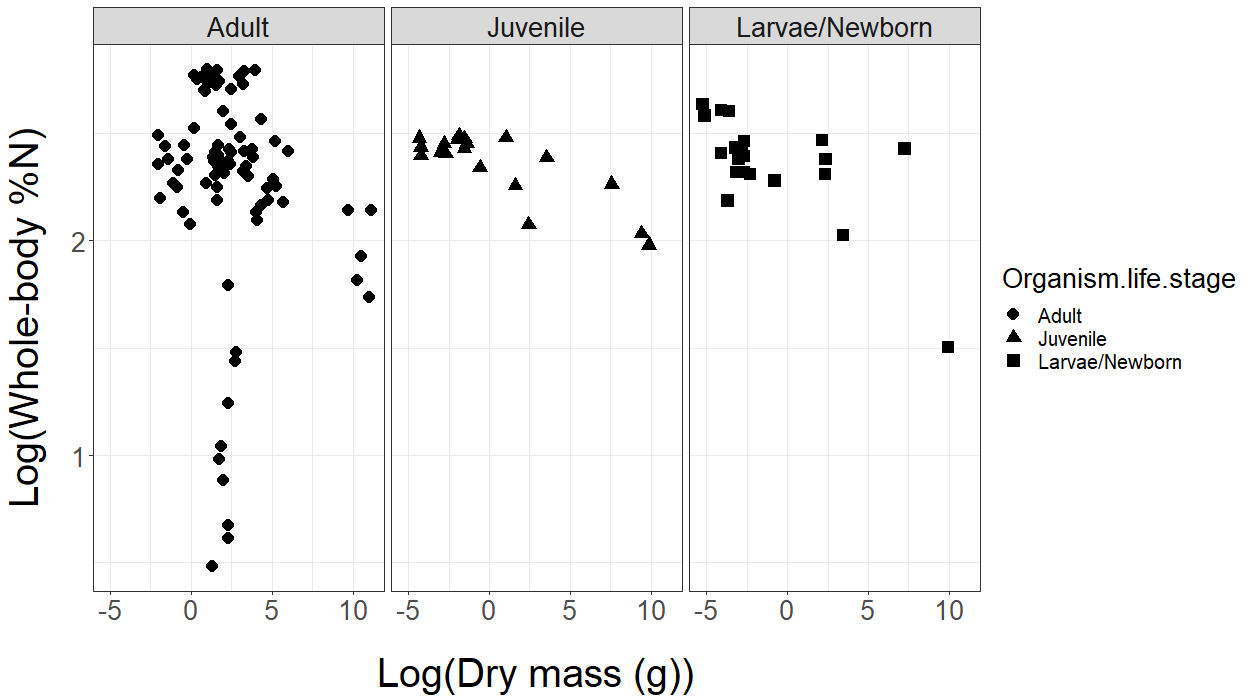


**Figure S23**: Effect of dry mass on whole-body %N (both transformed by log_e_), facetted by life stage.


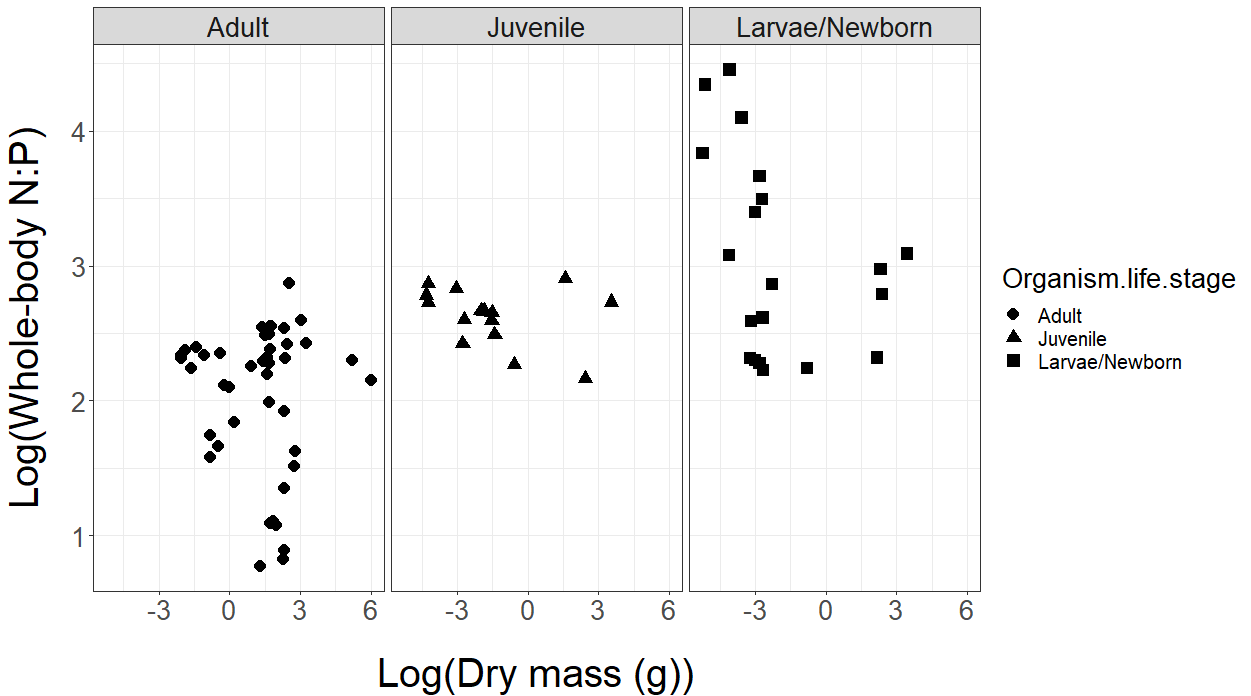


Figure S24: Effect of dry mass on whole-body N:P (both transformed by log_e_), facetted by life stage.
