# Supplementary material for: Life stage and taxonomy the most important factors determining vertebrate stoichiometry: A meta‐analysis
Source: Ecol Evol. 2022 Oct 1;12(10):e9354. doi: 10.1002/ece3.9354 (PMC9526032; doi:10.1002/ece3.9354)
Supplement: Supplementary file 2 — Appendix S2 [file ECE3-12-e9354-s001.docx]

## Appendix S2: DATA SOURCES

Allen, M. E., & Oftedal, O. (1993). Effect of dietary calcium concentration on mineral composition of fox geckos (*Hemidactylus garnoti*) and Cuban tree frogs (*Osteopilus septentrionalis*). *Journal of Zoo and Wildlife Medicine*, *24*(2), 118–128.

Andrieux, B., Signor, J., Guillou, V., Danger, M., & Jabot, F. (2021). Body stoichiometry of heterotrophs: Assessing drivers of interspecific variations in elemental composition. *Global Ecology and Biogeography*, *30*(4), 883–895. <https://doi>.org/10.1111/geb.13265

Atkinson, C. L., Golladay, S. W., & Smith, L. L. (2017). Larval anuran stable isotope signatures and stoichiometry across multiple geographically isolated wetlands in the Southeastern United States. *Southeastern Naturalist*, *16*(1), 87–104. <https://doi>.org/10.1656/058.016.0107

Bondi, C. A., Beier, C. M., Fierke, M. K., & Ducey, P. K. (2019). The role of feeding strategy in the tolerance of a terrestrial salamander (*Plethodon cinereus*) to biogeochemical changes in northern hardwood forests. *Canadian Journal of Zoology*, *97*(4), 281–293. <https://doi>.org/10.1139/cjz-2017-0302

Bouchard, S. S., & Bjorndal, K. A. (2000). Sea turtles as biological transporters of nutrients and energy from marine to terrestrial ecosystems. *Ecology*, *81*(8), 2305–2313. <https://doi>.org/10.2307/177116

Bouchard, S. S., & Bjorndal, K. A. (2006). Ontogenetic diet shifts and digestive constraints in the omnivorous freshwater turtle Trachemys scripta. *Physiological and Biochemical Zoology*, *79*(1), 150–158. <https://doi>.org/10.1086/498190

Brenes-Soto, A., Dierenfeld, E. S., Bosch, G., Hendriks, W. H., & Janssens, G. P. J. (2019). Gaining insights in the nutritional metabolism of amphibians: Analyzing body nutrient profiles of the African clawed frog, *Xenopus laevis*. *PeerJ*, *7*, e7365–e7365. <https://doi>.org/10.7717/peerj.7365

Burton, T. M., & Likens, G. E. (1975). Energy flow and nutrient cycling in salamander populations in the Hubbard Brook Experimental Forest, New Hampshire. *Ecology*, *56*(5), 1068–1080.

Capps, K. A., Berven, K. A., & Tiegs, S. D. (2015). Modelling nutrient transport and transformation by pool-breeding amphibians in forested landscapes using a 21-year dataset. *Freshwater Biology*, *60*(3), 500–511. <https://doi>.org/10.1111/fwb.12470

Cosgrove, J. J., Beermann, D. H., House, W. A., Toddes, B. D., & Dierenfeld, E. S. (2002). Whole-body nutrient composition of various ages of captive-bred bearded dragons (*Pogona vitteceps*) and adult wild anoles (*Anolis carolinensis*). *Zoo Biology*, *21*(5), 489–497. <https://doi>.org/10.1002/zoo.10055

Costello, D. M., & Michel, M. J. (2013). Predator-induced defenses in tadpoles confound body stoichiometry predictions of the general stress paradigm. *Ecology*, *94*(10), 2229–2236.

Dierenfeld, E. S. (n.d.). *Elemental content of free ranging lizards from Guam.* Unpublished dataset.

Duke, G. E., Ciganek, J. G., & Evanson, O. A. (1973). Food consumption and energy, water, and nitrogen budgets in captive great-horned owls (*Bubo virginianus*). *Comparative Biochemistry and Physiology*, *44*(A), 283–292.

Fritz, K. A., & Whiles, M. R. (2018). Amphibian-mediated nutrient fluxes across aquatic–terrestrial boundaries of temporary wetlands. *Freshwater Biology*, *63*(10), 1250–1259. <https://doi>.org/10.1111/fwb.13130

González, A. L., Fariña, J. M., Kay, A. D., Pinto, R., & Marquet, P. A. (2011). Exploring patterns and mechanisms of interspecific and intraspecific variation in body elemental composition of desert consumers. *Oikos*, *120*(8), 1247–1255. <https://doi>.org/10.1111/j.l

Grimshaw, A. H. M., Ovington, J. D., Betts, M. M., & Gibb, J. A. (1958). The mineral content of birds and insects in plantations of *Pinus silvestris* L. *Oikos*, *9*(1), 26–34.

Guariento, R. D., Carneiro, L. S., Jorge, J. S., Borges, A. N., Esteves, F. A., & Caliman, A. (2015). Interactive effects of predation risk and conspecific density on the nutrient stoichiometry of prey. *Ecology and Evolution*, *5*(21), 4747–4756. <https://doi>.org/10.1002/ece3.1740

Hood, W. R., Oftedal, O. T., & Kunz, T. H. (2006). Variation in body composition of female big brown bats (*Eptesicus fuscus*) during lactation. *Journal of Comparative Physiology B: Biochemical, Systemic, and Environmental Physiology*, *176*(8), 807–819. <https://doi>.org/10.1007/s00360-006-0102-y

Horn, S., & de la Vega, C. (2016). Relationships between fresh weight, dry weight, ash free dry weight, carbon and nitrogen content for selected vertebrates. *Journal of Experimental Marine Biology and Ecology*, *481*, 41–48. <https://doi>.org/10.1016/j.jembe.2016.04.010

Kirschman, L. J., Haslett, S., Fritz, K. A., Whiles, M. R., & Warne, R. W. (2016). Influence of physiological stress on nutrient stoichiometry in larval amphibians. *Physiological and Biochemical Zoology*, *89*(4), 313–321. <https://doi>.org/10.1086/687047

Liess, A., Rowe, O., Guo, J., Thomsson, G., & Lind, M. I. (2013). Hot tadpoles from cold environments need more nutrients—Life history and stoichiometry reflects latitudinal adaptation. *Journal of Animal Ecology*, *82*(6), 1316–1325. <https://doi>.org/10.1111/1365-2656.12107

Milanovich, J. R. (2016). *Elemental content of* Plethodon cinereus *from Indiana*. Unpublished dataset.

Milanovich, J. R., & Hopton, M. E. (2014). Stoichiometry of a semi-aquatic plethodontid salamander: Intraspecific variation due to location, size and diet. *Integrative Zoology*, *9*(5), 613–622. <https://doi>.org/10.1111/1749-4877.12114

Milanovich, J. R., & Peterman, W. E. (2016). Revisiting Burton and Likens (1975): Nutrient standing stock and biomass of a terrestrial salamander in the Midwestern United States. *Copeia*, *104*(1), 165–171. <https://doi>.org/10.1643/ot-14-180

Norlin, L., Byström, P., Karlsson, J., Johansson, M., & Liess, A. (2016). Climate change will alter amphibian-mediated nutrient pathways: Evidence from *Rana temporaria* tadpoles in experimental ponds. *Freshwater Biology*, *61*(4), 472–485. <https://doi>.org/10.1111/fwb.12720

Prater, C., Scott, D. E., Lance, S. L., Nunziata, S. O., Sherman, R., Tomczyk, N., Capps, K. A., & Jeyasingh, P. D. (2019). Understanding variation in salamander ionomes: A nutrient balance approach. *Freshwater Biology*, *64*(2), 294–305. <https://doi>.org/10.1111/fwb.13216

Randolph, A. J. C., Cameron, G. N., & Mcclure, P. A. (1995). Nutritional requirements for reproduction in the Hispid cotton rat, *Sigmodon hispidus*. *Journal of Mammalology*, *76*(4), 1113–1126.

Register, K. J., & Whiles, M. R. (2006). Decomposition rates of salamander (*Ambystoma maculatum*) life stages and associated energy and nutrient fluxes in ponds and adjacent forest in Southern Illinois. *Copeia*, *2006*(4), 640–649. <https://doi>.org/10.1643/0045-8511(2006)6[640:drosam]2.0.co;2

Rizzuto, M., Leroux, S. J., Vander Wal, E., Wiersma, Y. F., Heckford, T. R., & Balluffi-Fry, J. (2019). Patterns and potential drivers of intraspecific variability in the body C, N, and P composition of a terrestrial consumer, the snowshoe hare (*Lepus americanus*). *Ecology and Evolution*, *9*(24), 14453–14464. <https://doi>.org/10.1002/ece3.5880

Schairer, M. L., Dierenfeld, E. S., & Fitzpatrick, M. P. (1998). Nutrient Composition of Whole Green Frogs, *Rana clamitans* and Southern Toads, *Bufo terrestris*. *Bulletin of the Association of Reptilian and Amphibian Veterinarians*, *8*(3), 17–20. <https://doi>.org/10.5818/1076-3139.8.3.17

Semlitsch, R. D., O’Donnell, K. M., & Thompson, F. R. (2014). Abundance, biomass production, nutrient content, and the possible role of terrestrial salamanders in Missouri Ozark forest ecosystems. *Canadian Journal of Zoology*, *92*(12), 997–1004. <https://doi>.org/10.1139/cjz-2014-0141

Stephens, J. P., Berven, K. A., & Tiegs, S. D. (2013). Anthropogenic changes to leaf litter input affect the fitness of a larval amphibian. *Freshwater Biology*, *58*(8), 1631–1646. <https://doi>.org/10.1111/fwb.12155

Sterrett, S. C., Maerz, J. C., & Katz, R. A. (2015). What can turtles teach us about the theory of ecological stoichiometry? *Freshwater Biology*, *60*(3), 443–455. <https://doi>.org/10.1111/fwb.12516

Sturges, F. W., Holmes, R. T., & Likens, G. E. (1974). The role of birds in nutrient cycling in a northern hardwoods ecosystem. *Ecology*, *55*(1), 149–155.

Tabaka, C. S., Ullrey, D. E., Sikarskie, J. G., DeBar, S. R., & Ku, P. K. (1996). Diet, cast composition, and energy and nutrient intake of red-tailed hawks (*Buteo jamaicensis*), great horned owls (*Bubo virginianus*), and turkey vultures (*Cathartes aura*). *Journal of Zoo and Wildlife Medicine*, *27*(2), 187–196.

Tiegs, S. D., Berven, K. A., Carmack, D. J., & Capps, K. A. (2016). Stoichiometric implications of a biphasic life cycle. *Oecologia*, *180*(3), 853–863. <https://doi>.org/10.1007/s00442-015-3504-2

Vanni, M. J., Flecker, a S., Hood, J. M., & Headworth, J. L. (2002). Stoichiometry of nutrient recycling by vertebrates in a tropical stream: Linking biodiversity and ecosystem function. *Ecology Letters*, *5*, 285–293. <https://doi>.org/10.1046/j.1461-0248.2002.00314.x

Whatley, C., Tapley, B., Chang, Y., Newton-Yowens, J., McKendry, D., & Michaels, C. (2020). Impacts of UVB provision on serum vitamin D3, pigmentation, growth rates and total body mineral content in Mallorcan midwife toad larvae (*Alytes muletensis*). *Journal of Zoo and Aquarium Research*, *8*(1), 37–44. <https://doi>.org/10.19227/jzar.v8i1.434

Wu, D., Deng, X., & Xu, Y. (1991). Standing crops of elements and atomic ratios for a small mammal community captured by snap-traps in the Ailao Mountains. *Zoological Research*, *12*(2), 187–192.
